# FishNET: An automated relational database for zebrafish colony management

**DOI:** 10.1371/journal.pbio.3000343

**Published:** 2019-06-20

**Authors:** Abiud Cantu Gutierrez, Manuel Cantu Gutierrez, Alexander M. Rhyner, Oscar E. Ruiz, George T. Eisenhoffer, Joshua D. Wythe

**Affiliations:** 1 Cardiovascular Research Institute, Baylor College of Medicine, Houston, Texas, United States of America; 2 Department of Molecular Physiology and Biophysics, Baylor College of Medicine, Houston, Texas, United States of America; 3 Graduate Program in Developmental Biology, Baylor College of Medicine, Houston, Texas, United States of America; 4 Department of Genetics, MD Anderson Cancer Center, Houston, Texas, United States of America; Karolinska Institutet, SWEDEN

## Abstract

The zebrafish *Danio rerio* is a powerful model system to study the genetics of development and disease. However, maintenance of zebrafish husbandry records is both time intensive and laborious, and a standardized way to manage and track the large amount of unique lines in a given laboratory or centralized facility has not been embraced by the field. Here, we present FishNET, an intuitive, open-source, relational database for managing data and information related to zebrafish husbandry and maintenance. By creating a “virtual facility,” FishNET enables users to remotely inspect the rooms, racks, tanks, and lines within a given facility. Importantly, FishNET scales from one laboratory to an entire facility with several laboratories to multiple facilities, generating a cohesive laboratory and community-based platform. Automated data entry eliminates confusion regarding line nomenclature and streamlines maintenance of individual lines, while flexible query forms allow researchers to retrieve database records based on user-defined criteria. FishNET also links associated embryonic and adult biological samples with data, such as genotyping results or confocal images, to enable robust and efficient colony management and storage of laboratory information. A shared calendar function with email notifications and automated reminders for line turnover, automated tank counts, and census reports promote communication with both end users and administrators. The expected benefits of FishNET are improved vivaria efficiency, increased quality control for experimental numbers, and flexible data reporting and retrieval. FishNET’s easy, intuitive record management and open-source, end-user–modifiable architecture provides an efficient solution to real-time zebrafish colony management for users throughout a facility and institution and, in some cases, across entire research hubs.

## Abstract 2

With the continued growth of zebrafish as a genetic model system, the ability to robustly catalogue and monitor the animals within a zebrafish colony is critical. This paper presents an open-source, non-coding–based, relational database solution that streamlines data entry, saves valuable time, and enables easy real-time monitoring and analysis of colony health and fecundity.

## Author summary

FishNET facilitates remote tracking of individual zebrafish lines and links associated biological resources to enable robust and efficient colony management and storage of laboratory information related to zebrafish.

## Introduction

The fecundity, rapid development, external fertilization, amenability to both forward [1–7] and reverse [8–11] genetic approaches, conservation of core vertebrate protein-coding genes [12], small size, and inexpensive husbandry costs make zebrafish a powerful vertebrate model for studying human physiology and disease [13, 14]. Moreover, their optical transparency [15–17] and readily available suite of genetic tools (Tol1 and 2 [transposable element] tranposase-mediated transgenesis, Gal4/UAS [yeast Gal4 transcriptional activator/upstream activation sequence] gene expression system, Cre/loxP [bacterial Cre recombinase gene/locus of X(cross)-over in P1] genetics, etc.) [18–27] and fluorescent reporters (e.g., gCaMP [green fluorescent protein, calmodulin, and M13 peptide], lifeact fluorescent proteins, etc.) [18, 28] have made zebrafish the premier model system for studying vertebrate biology in real time. These features, combined with their impressive regenerative capacity, also make zebrafish ideal for studying vertebrate tissue and organ regeneration [29, 30]. Given these advantages, it is no wonder that fields as diverse as developmental neurobiology [31] and cancer [32] have leveraged the embryonic and adult zebrafish, respectively, to make valuable biological insights. As more and more labs embrace this model system and shared-use zebrafish “core” facilities continue to be built across the world, there has been an explosion in the amount of transgenic and mutant lines generated by the community, creating a significant need for accurate, automated, real-time colony management tracking software. This need for a centralized database is not only desirable at the level of an individual laboratory group but in some cases is necessary throughout a facility and institution or even across an entire research hub as researchers desire to build a more cohesive zebrafish community.

Despite the importance of effective data management in animal research, many investigators employ handwritten notebooks or spreadsheet applications for managing small and medium-sized animal colonies. While these ad hoc data entry approaches offer the benefit of being simple to adopt, they do not scale with increased user numbers (even within a single laboratory), and they are not practical across an entire facility. Relying upon individuals to enter the correct line nomenclature (e.g., allele) and accurate information (e.g., sex, date of birth, etc.) unnecessarily exposes these “databases” to avoidable operator error. Additionally, paper records have the significant drawback that they cannot be accessed simultaneously by multiple users, they cannot be viewed remotely, and they can be misplaced or destroyed. On the other hand, electronic spreadsheet databases suffer from their own limitations, as multiple users cannot access them simultaneously in real-time. In addition, data stored in this format are challenging to mine; they typically lack a uniform/controlled lexicon, and best practice rules for data entry are absent, thus making them highly susceptible to operator error. Moreover, simple spreadsheets (e.g., Excel) cannot navigate complex data structures, such as breeding schemes or pedigrees, as required in a model colony management software.

Paraphrasing Silver [33] and as epitomized in the Jax Colony Management Software for mice [34], an ideal database would track (1) individual animals and their ancestors, (2) matings between animals, (3) progeny born from such matings (e.g., litters) and the individuals within litters that are used in experiments, and (4) experimental materials (tissues and DNA samples) obtained from individual animals. Such a database should also format records so that determination of relationships between any components of the colony is possible (e.g., which tissue came from which animal). Ideally, this platform would feature an intuitive graphical user interface, have a robust online community to support trouble shooting or modifications to the underlying architecture or functionality, and also be open source.

A handful of software solutions specifically designed for managing zebrafish animal husbandry exist. A summary of some of the currently available zebrafish colony management applications, both commercial and open source, is provided in Table 1, while their functionalities are summarized in Table 2. Available solutions for data management also vary significantly in terms of cost, implementation requirements, flexibility, and level of user support. Additionally, few of these options are open source, and even fewer also record phenotyping and experimental data. These drawbacks, along with the cost of commercial options or the potentially intimidating learning curve associated with adopting open-source options—such as creation and maintenance of structured query language (SQL) databases, along with knowledge of programming languages like Python or personal home page (PHP) to manage them—may explain the reluctance of the zebrafish community to embrace these solutions.

**Table 1:** Examples of currently available zebrafish colony management applications.

Application	URL	License Fee	Operating System	Data Storage
**ZeBase**	http://zebase.bio.purdue.edu/	Free (open source)	Any device with web browser (PC, Mac, Unix/Linux)	Web server running on a personal computer or dedicated server
**Zebrabase**	https://public.zebrabase.org/	Free	Any device with web browser (PC, Mac, Unix/Linux)	Web server running on a personal computer or dedicated server
**DanioData**	www.daniodata.com	Free (first rack?) for app, but unclear about server issues/fees	iPad Application	
**PyRAT Aquatic**	https://www.scionics.com/pyrat_aquatic.html	Fee (request quote)	Any device with web browser (PC, Mac, Unix/Linux)	Full license purchase or hosted solutions available
**Facile Fish**	https://zebrafish.jimdo.com	Free (open source)	Any device with web browser (PC, Mac, Unix/Linux)	Web Server running on a personal window computer or dedicated server running PHP/Apache
**Fish Database**	http://aqacs.uoregon.edu/zf/files/	Free (FMPA)	Windows 10, 9, 8, 7 with latest updates; macOS High Sierra 10.13 or Sierra 10.12	Variable to dedicated computer storage capabilities

**Abbreviations**: FMPA, FileMaker Pro Advanced; PHP, XXX; PyRAT, Python-based Relational Animal Tracking.

**Table 2:** Features of currently available zebrafish colony management applications.

App	View Pedigree	Create Resources	Track Matings	Virtual Facility	Dead Fish Report	Calendar Reminders	Reusable Labels	Real-Time Graphical Reports	Water Quality Reports	Annual IACUC Reports
**ZeBase**	**X**	**X**	**X**	**X**	**✓**	**X**	**X**	**✓**	**✓**	**X**
**DanioData**	**X**	**✓**	**X**	**✓**	**✓**	**✓**	**✓**	**✓**	**✓**	**X**
**PyRAT** **Aquatic**	**✓**	**X**	**✓**	**X**	**✓**	**X**	**✓**	**✓**	**X**	**X**
**Facile** **Fish**	**✓**	**X**	**✓**	**X**	**✓**	**✓**	**X**	**X**	**✓**	**X**
**Fish** **Database**	**X**	**X**	**✓**	**X**	**✓**	**X**	**✓**	**X**	**X**	**X**
**ZebraBase**	**✓**	**✓**	**✓**	**✓**	**X**	**✓**	**✓**	**✓**	**X**	**X**
**FishNET**	**✓**	**✓**	**✓**	**✓**	**✓**	**✓**	**✓**	**✓**	**✓**	**✓**

**Abbreviations**: IACUC, Institutional Animal Care and Use Committee; PyRAT, Python-based Relational Animal Tracking.

Here, we describe FishNET. FishNET is a comprehensive relational database application developed specifically to meet biomedical research community demands for a well-engineered, flexible database system supporting zebrafish animal husbandry and data management. FishNET meets the aforementioned criteria for an ideal record-keeping system with several added benefits, including a remotely accessible virtual facility view; a pedigree function; barcode-scannable labels for tanks, crosses, embryos, and fry; a calendar function that emails users for events such as line turnover or graduation of fry to the nursery; sick fish reports; records for genotyping protocols; and real-time records of all fish tanks, their use, and water quality in a facility for billing purposes and Institutional Animal Care and Use Committee (IACUC) reporting purposes.

FishNET runs directly on Macintosh or PC computers using the FileMaker Pro Advanced (FMPA) application. Hosted databases can also be viewed remotely via web browsers on PC, Macintosh, or Linux computers by incorporating FileMaker WebDirect. Furthermore, it can be accessed on iPad and iPhones through the FileMaker Go application in the App Store (https://apps.apple.com/us/app/filemaker-go-18/id1438460792?ls=1). The functional features of FishNET include support for controlled vocabularies (such as uniform allele nomenclature to avoid confusion regarding the origin of a transgenic or mutant line), multiuser capabilities, quick and accurate data reporting, pedigree tracking, animal husbandry workflow, sample tracking, and experimental data capture. The expected benefits of FishNET are improved vivaria efficiency, increased quality control for experimental numbers, and flexible data reporting and retrieval. FishNET is a freely available tool, and FileMaker software is a well-supported application with a large user base. Free trial versions of FMPA 17 are available at https://content.filemaker.com/filemaker-trial-en-rf. FileMaker Go (for iOS mobile devices) is available at the App Store (https://www.filemaker.com/products/filemaker-go/). A full database, as well as an empty shell, of FishNET is freely available at http://www.wythelab.com/wythe-lab-databases (or via S1 File).

## Materials and methods

### Ethics statement

Zebrafish protocols were approved by the IACUC at Baylor College of Medicine and the University of Texas MD Anderson Cancer Center.

### System architecture

FishNET employs a normalized relational database that can be run through FMPA. The relational data model that underlies FishNET minimizes data redundancy, enforces data integrity, leverages controlled vocabularies to ensure proper allele designations, and enables accurate data retrieval against large data sets. The user interface enables addition of new functionality and enhancement of existing functions without major modifications to the basic infrastructure, enabling easy adaptation to user-specific needs. FMPA ensures safe multiuser concurrent data entry and editing in real time. Additionally, FishNET features a built-in barcode generator and barcode reader, and when these features are combined with a validated printer (as we demonstrate herein), together, they provide instant recognition and tracking of all fish stock information and related actions on a mobile device.

**Fig 1:**
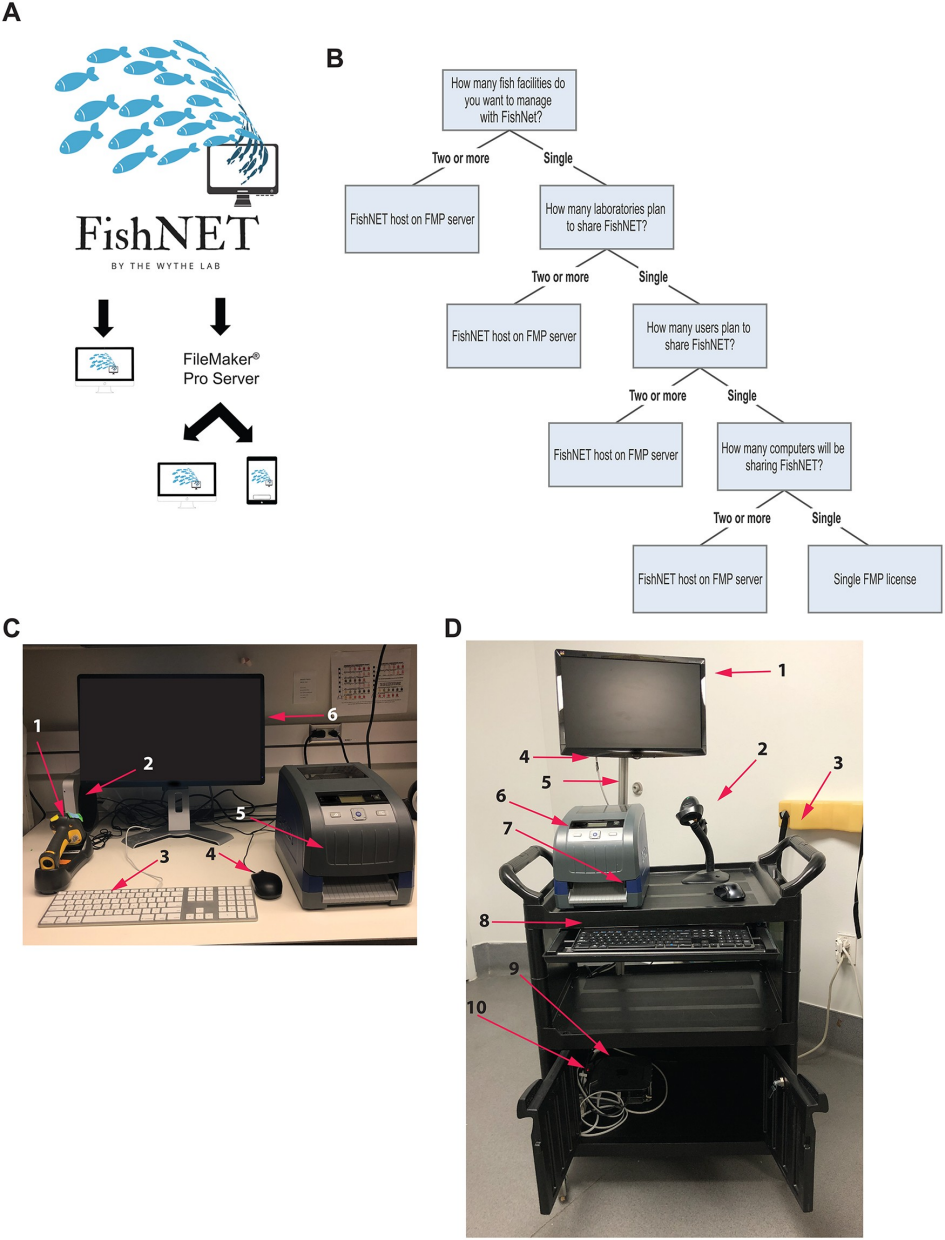
Schematic representation of FishNET configuration. (A) FishNET can be used as a single unit installed in one PC alone or through a server that can support multiple computers and handheld devices. (B) Decision tree to determine if a single FMPA License is needed or multiple licenses hosted on a server are required. (C) The stationary, standalone desktop configuration for running FishNET consists of 1) barcode scanner, 2) Mac Mini, 3) keyboard, 4) mouse, 5) BBP33 Brady printer (Milwaukee, WI, USA), and 6) computer monitor. (D) The mobile configuration for FishNET consists of 1) 19” widescreen LED backlight LCD monitor, 2) barcode scanner, 3) dual-handle plastic enclosed shelf service cart, 4) DisplayPort to VGA adapter converter cable, 5) V-slot extruded aluminum stand for the monitor, 6) BBP33 Brady printer, 7) wireless mouse (far right, on top of cart), 8) a wireless waterproof keyboard (in cart drawer) 9) Mac Mini with, and 10) a Mac Mini security mount (stored in cart cabinet behind lockable doors). Not shown in the lower cabinet are the surge protector outlet power strip and an uninterruptible power supply (e.g., an APC UPS from Schneider Electric; West Kingston, RI, USA) to make the entire unit mobile. FMPA, FileMaker Pro Advanced; LCD, liquid crystal display; LED, light-emitting diode; VGA, visual graphics array.

An overview of the two primary configurations of FishNET is shown in Fig 1A. FishNET can be set up to run as a standalone database on a personal computer running a Windows or MacOS operating system. Alternatively, FishNET can be hosted on a central computer running a Macintosh or Windows operating system using FMPA Server software to support small- to medium-sized research groups in which FMPA client software is installed on all user desktop and laptop computers (e.g., 1 license of FMPA per accessing computer). This same FMPA Server configuration also supports use of FishNET through the FileMaker Go App on mobile handheld devices with barcode readers running iOS 11.2 or later (e.g., iPad, iPhone). Handheld computers that have been tested include iPad Air 2 as well as iPhone 6s and iPhone X. In the server configuration, FishNET can be accessed—with appropriate user authentication capabilities—on any internet-capable device running Safari, Chrome, Internet Explorer, or Microsoft Edge internet browsers using FileMaker WebDirect.

The cost of setting up and hosting FishNET will vary depending on the configuration. The number of users and their need to modify the underlying database architecture (e.g., do they need to function as an administrator or simply upload, access, and download data) will dictate whether a standalone license is sufficient or whether multiple licenses that each connect to an FMPA Server (hosted via an on-premise server) or FileMaker Cloud (hosted remotely via FileMaker, not described here) are required. At this time, an annual FMPA license costs $540 for 5 licenses per year (academic pricing) with FMPA Server included, while a permanent individual instance of FMPA costs $324 and FileMaker Cloud costs $400 (user licenses not included). The cost to host FMP Server will vary by institution and will also depend on whether the institution hosts it on their own server (which requires Windows Server 2016 or newer) or if it is instead hosted locally by the laboratory (for instance, on a computer running MacOS Sierra 10.12 [or newer] software). A guide to determining which license is appropriate for your group is provided in Fig 1B, as are images of the stationary and mobile stations in Fig 1C and 1D, respectively. A more detailed comparison between the costs of FMP Server and Cloud is provided in S1 Table.

### Hardware requirements

The base hardware requirements of FMPA and FishNET are relatively modest. FishNET runs smoothly on a late 2014 Mac mini with a 2.6 GHz, dual-core Intel i5 (2278U) processor with 8 GB of 1,600 MHz LPDDR3 onboard memory, 3 MB virtual memory, and a 1-TB hard drive and 802.11ac Wi-Fi wireless networking and Bluetooth 4.0 wireless technology requiring 100–240V AC. While the database itself can be set up multiple ways, below, we describe two standard configurations. One option is an immobile station on a bench top within the facility room that requires a wireless barcode reader integrated with the computer (to read barcodes and enter data) as well as a label printer (for the racks, tanks, crosses, and fry) (Fig 1C). For multiple users, a more sensible configuration may be to locally host FishNET (this requires an FMPA Server license, which is included in the purchase of a team FMPA license—which covers 5 or more individual licenses). This set-up allows for simultaneous, multiuser access and data entry using either FMPA on the same network or through an internet web browser on the same network via FileMaker WebDirect. In this configuration, access on the same network could also be achieved on a handheld device using the FileMaker Go application (which is restricted to iPads and iPhones running iOS 11.2 or above). Individual licenses for FMPA would only be required for users that want to easily print labels with barcodes and run the automated genotyping annotation script (see below) or those that need to modify the underlying database architecture. The hardware and costs for this configuration are outlined in Table 3.

**Table 3:** Hardware required for the immobile, desktop FishNET configuration.

Item	Use	Cat #	Manufacturer	Cost (Vendor)
**Mac Mini security mount**	To immobilize the Mac Mini computer/security	MMEN76	Maclocks.com	$49.95 (Maclocks.com)
**Mac Mini**	Computer	MGEN2LL/A	Apple	$699 (Apple)
**19" widescreen LED backlight LCD monitor**	Visualization	VA1938WA-LED	ViewSonic	$102 (CDW)
**6 outlet, 6 ft, power strip with surge protector**	Power	Super 7 Protect it! Surge protector S9TLP606	Tripp Lite	$14.40 (officemax)
**Bluetooth, waterproof keyboard**	Data entry	WKB-2000BW (for Mac)	Adesso	$29.95 (Amazon)
**Wireless mouse**	Data entry, navigation	M510 mouse, 910–001822	Logitech	$38.99 (CDW)
**Wireless, waterproof barcode scanner**	Barcode scanning, data entry	19 140 592	Adesso	$125 (newegg.com)
**BBP33 W/AUTO CUT PRINTER**	Print Labels	9SIAFJ86WS6511	Brady	$1,169.33 (Fisher Scientific)
**RIBBON, R6200 BLK, 4.33 X200FT**	Ribbon needed to print labels	19 140 590	Brady	$48.72 (Fisher Scientific)
**Direct custom labels 1,000**	Cling labels	NC1568023	Brady	$469.26 (Fisher Scientific)
**Optional: iPad 32 GB, Wi-Fi only, fifth Generation**	Data entry, visualization	MP2F2LL/A	Apple	$279 (Target)

**Abbreviations**: LCD, liquid crystal display; LED, light-emitting diode.

A second configuration is for a mobile station that can travel throughout an entire facility (Fig 1D). The costs and components of this set-up are shown in Table 4. A basic overview comparison between the computing software and hardware costs for all FishNET configurations is provided in Table 5.

**Table 4:** Hardware required for the mobile FishNET configuration.

Application	Use	Cat #	Manufacturer	Cost (Vendor)
**Dual-handle plastic enclosed shelf service cart**	For Mac Mini and peripherals	#940Z122	Gamut	$447.19 (Gamut.com)
**Backup portable battery**	Battery for Mobility	APC-Back UPS PRO Pro BR1000G	APC	$133 (Amazon)
**Rocketfish 13”–26" tilting low-profile TV wall mount**	To mount PC monitor to aluminum rod	Rf-tvmlpto1v2	Rocketfish	$21 (ebay)
**V-slot extruded aluminum 20 × 20 × 1,500**	To hold PC monitor to cart	AAA-019	Hobby-Fab	$17.69 (ebay)
**6 ft mDP to VGA adapter converter cable—mDP to VGA 1,920 × 1,200**	To wire Mac Mini to PC monitor	MDP2VGAMM6W	StarTech	$41.99 (StarTech.com)

**Abbreviations**: mDP, mini DisplayPort; VGA, visual graphics array.

**Table 5:** Hardware, software, and cost comparisons between FishNET configurations.

Application	Numbers of Users Supported	Hardware Requirements	Platform	Contract Length	Cost
**FMPA (1 instance)**	1 Computer	For computer running Windows: CPU, 1 GHz or faster x86- or x64-bit processor; RAM, 1 GB For computers running macOS: RAM, 2 GB	Windows 10, 9, 8, 7 with latest updates. macOS High Sierra 10.13 or Sierra 10.12	Single permanent license	$324
**FMPA (for teams of 5 or more)**	Allows for 5 simultaneous client connections to one or multiple databases	Same as above	Same as above	Billed annually or permanent license	$540 if billed annually, $1,620 for 5 permanent licenses
**FileMaker Server 17**	Allows up to 500 users	CPU: dual core RAM: 8 GB Hard drive: 80 GB or more	Windows Server 2016 Standard Edition (with Desktop Experience), Windows Server 2012 R2 with Update, macOS High Sierra 10.13 or MacOS Sierra 10.12.	Included with FMP Advanced Team License	Variable
**FileMaker** **Cloud 1.17**	Allows up to 100 users	NA	NA	Billed annually	$400 (user licenses not included)
**FileMaker Go**	NA	iPad or iPhone	iOS 11.2 or above	NA	Free (Apple App Store)

**Abbreviations**: FMPA, FileMaker Pro Advanced; NA, not applicable.

### Software requirements

Regardless of the hardware set-up, the main drawback of using a commercial relational database platform is that while it is open source, non-coding–based, and user modifiable, a facility or laboratory must purchase a permanent or annual license to use FMPA. Fortunately, several flexible options exist, as outlined below. Additionally, the FileMaker Go App is compatible with Apple mobile devices (e.g., iPhone and iPad). If FishNET will be hosted, then a computer with a Dual-Core CPU processor, 8 GB of RAM, and at least 80 GB of hard drive storage running Windows Server 2016 Standard Edition (with Desktop Experience), Windows Server 2012 R2 with Update, MacOS High Sierra 10.13, or MacOS Sierra 10.12 will be required. The FMP Server license is included when you obtain at least 5 licenses of FMPA, making this the logical choice for most user groups.

### Data security and backup

To protect the user’s data, FMPA Server offers AES-256 encryption. It also supports third-party secure sockets layer (SSL) certificates to establish secure links between the server and web browsers. Furthermore, using an institution’s wireless local area network (Wi-Fi) to transfer files from the FMPA Server to individual users adds another layer of security (provided that the institution requires user authentication to connect to the network/has a secure firewall).

A centralized database, such as FishNET, offers the advantage of simplifying data redundancy and mirroring (e.g., creating backups). When using a single instance of FMPA on a single computer, a user or lab can back up the entire contents of the hard drive, along with the FMPA database file(s), using a standard backup program (e.g., Time Machine, SuperDuper!, etc.), storing these copies on an external hard drive or remotely in the cloud. In the case of FMPA Server, the program by default creates a backup daily, storing the last seven backups (and successively rewriting over them from oldest to newest), with the option to keep specific backups at will. In this case, we suggest copying FishNET to an external drive.

## Results

### General overview, installing the database

We have developed a system for positional identification of tanks and lines based on user-defined “Facilities,” “Rooms,” “Racks,” “Tanks,” and “Lines.” The underlying infrastructure of this system is a relational database. A typical database is composed of tables of individual entries or “Records” (as they are referred to in FMPA) that contain data (in the case of FishNET, these data are information such as tank number, genotype, and date of birth). In a relational database, these records relate to one another through shared or common data (e.g., tank identifier number, genotype, etc.), and as such, these records (and all of their associated data) can be sorted, queried, and viewed independently or in a list format. Tables of records within FishNET can be broadly grouped into seven categories (or “layouts,” in FMPA terms): Tanks, Crosses, Lines, Harvests, Nursery, Labs, and Statistics. Information regarding the allele (Lines), parental strain(s)/mating history (Tanks and Crosses), progeny (Nursery), owner of the tank/IACUC number(s) associated with a tank (Labs and Tanks), usage (Harvests), and water quality/births/deaths (Statistics) are all stored in these various records, enabling robust and detailed records of zebrafish husbandry within a research group or across an entire facility. An eighth category, Virtual Facility, which contains unique identifiers for the facility, room, rack, row, and column of a given tank, creates a virtual facility containing room(s), rack(s), tank(s), and nursery of unique zebrafish lines, thus enabling users or administrators to view all occupied tanks within the facility (as well as the pertinent information for each individual tank: sex, age, genotype) and easily and efficiently track and locate tanks within a facility. A final category is the “Calendar” layout, which relates to all other tables (e.g., Crosses, Tanks, Nursery, etc.). This category has email functionality to create reminders for graduating fry to the main system, turning over lines, or any other user-defined event. Finally, each tank, cross, and fry/larvae, as well as each rack, is uniquely barcoded to enable, fast, reliable data entry and functionality (such as moving tanks within or across racks, setting up matings, recording dead fish, etc.).

**Fig 2:**
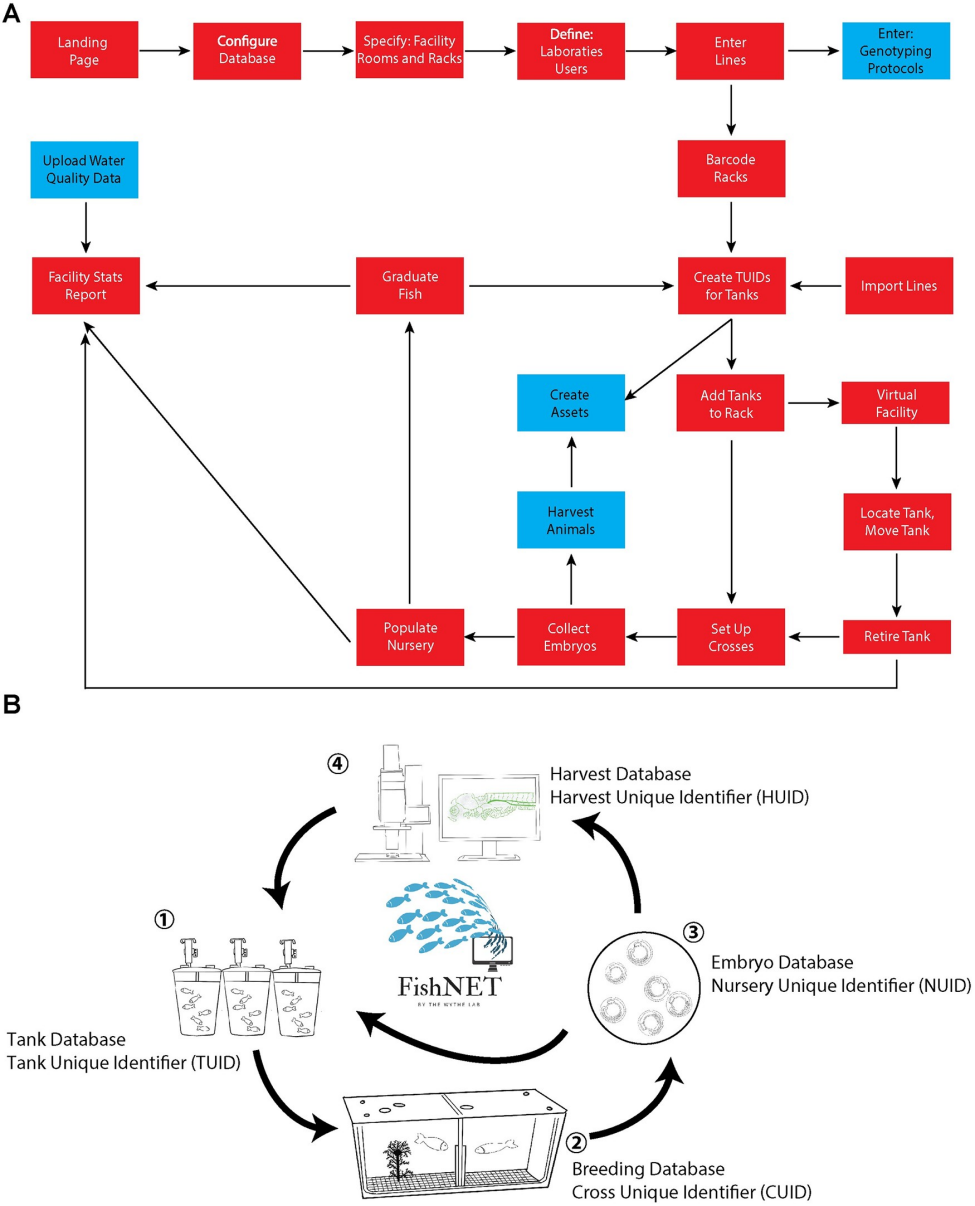
Step-by-step overview for implementing FishNET. (A) The logic flow for configuring the database is shown, with essential steps in red and optional steps in blue. Simplified relationships are indicated by the directional arrows. (B) An overview of the resources that FishNET can relate and manage. (1) A tank with adult zebrafish is given a TUID. If two adults are crossed to produce offspring (2), a CUID is assigned to the mating. Embryos are collected (3) and given an NUID, and they can either be processed for experimental processes (4) and be given an HUID or (1) graduated to the main system and given a new TUID. CUID, cross unique identifier; HUID, harvest unique identifier; NUID, nursery unique identifier; TUID, tank unique identifier.

Once you have determined the correct FMPA configuration for your needs (e.g., locally hosted server, cloud, etc. [Fig 1A and 1B]) and purchased and installed the software, visit http://www.wythelab.com/wythe-lab-databases and download the latest version of FishNET. If using a single standalone FMPA license, the user only needs to open the FishNETv1.fmp12 file and proceed with the set up. If using the FMPA Server, the downloaded file needs to be placed in the FMP Server database folder. In macOS, this folder is placed by default in /Library/FileMaker Server/Data/Databases, or in Windows, in C:/Program Files/FileMaker/FileMaker Server/Data/Databases. Below, we provide a logical, step-by-step guide for creating your virtual facility, entering in lines, and populating racks with these lines, an overview of which is provided in Fig 2A. An overview of how resources relate to one another within FishNET can be found in Fig 2B. In short, (1) a tank with adult zebrafish is given a tank unique identifier (TUID). If two adults are crossed to (2) produce offspring, a cross unique identifier (CUID) is assigned to the mating. Embryos resulting from this cross (3) are given a nursery unique identifier (NUID), and they can either be processed for experimental purposes (4) and be given a harvest unique identifier (HUID) or (1) graduated to the system and given a new TUID.

### Importing FishNET to FMP

**Fig 3:**
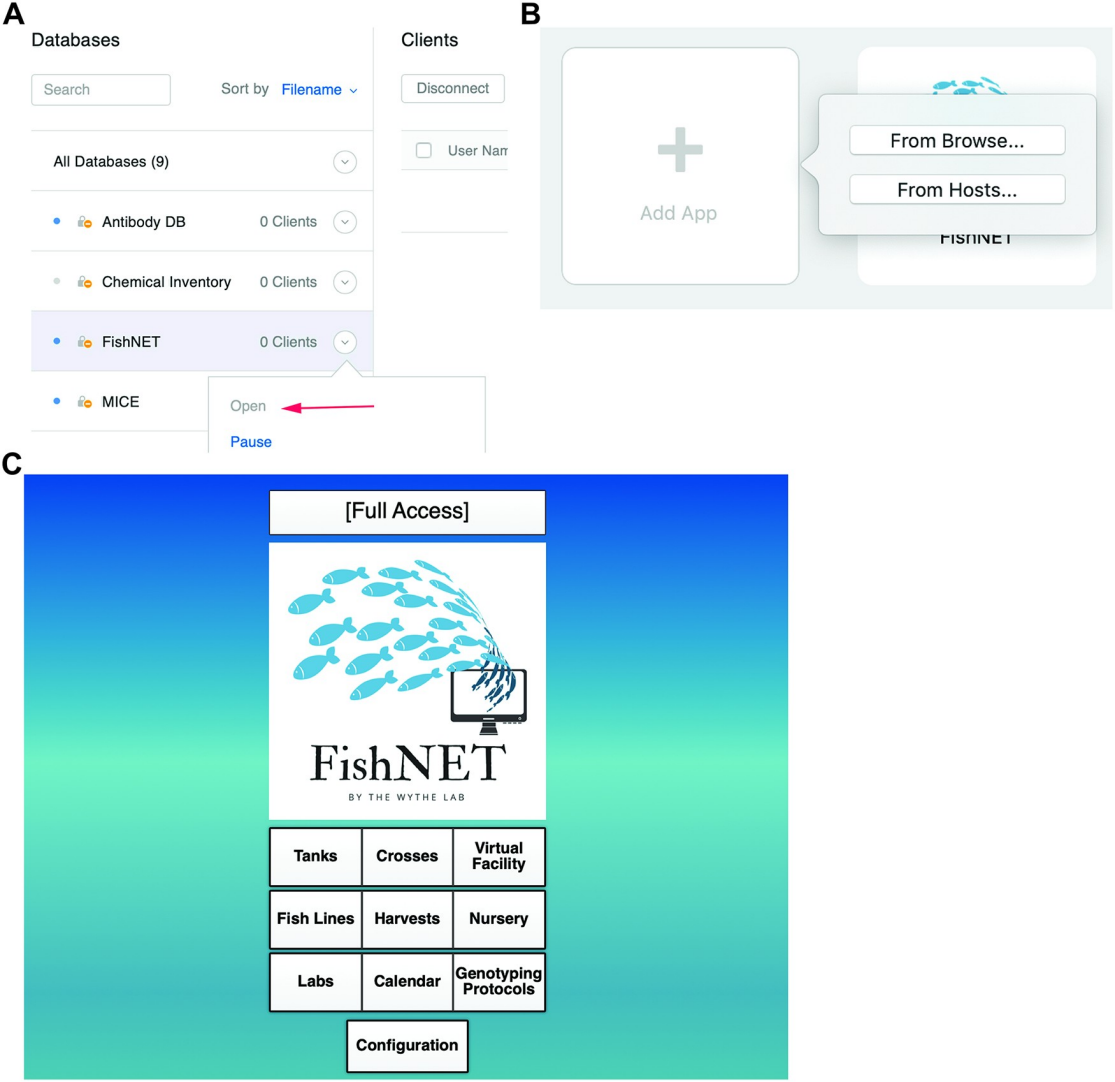
Importing FishNET to FMPA. (A) Activation of FishNET database from the FileMaker Server 17 administrator console can be done by selecting “Open” (indicated by red arrow). (B) FishNET App can be imported to FMPA locally by selecting “From Browser…” or from FileMaker Server selecting “From Hosts…” (indicated by red arrow). (C) FishNET landing page with full access privileges. FMPA, FileMaker Pro Advanced.

After depositing the downloaded file in the appropriate folder (as outline above), there needs to be a one-time activation in the FileMaker Server Admin console to bring FishNET online. This can be done by selecting the database and selecting “Open” (Fig 3A). In order to add the database to individual FMPA instances, users need to select “Add App From Hosts,” then add the host using the IP address of the server. This can be found in the FileMaker Server Admin console (Fig 3B).

### Creating an administrator user account

Upon opening the software, a window asking for username and password will appear. Enter “admin” as the username and “admin1234” as the password to access the database. Upon selecting sign in, the landing page should be visible (Fig 3C). Here, you will see a directory below the main FishNET icon. You can also select from the pulldown “Layout” menu in the upper left-hand corner for navigation. From the “Landing Page,” or “Home,” you will first select File/Manage…/Security. By default, an “admin user” is set up. If the database will be shared between different laboratories, we recommend setting up a password to access the administrator account because this account can access all records in the database and can also change the underlying architecture of each layout, whereas the individual labs will only see and modify records that belong to their respective laboratories and are prevented from modifying database-wide records (**see Video 1**: https://youtu.be/BwCD6-r6MM4). Creating labs and individual users will be addressed later.

### Creating facilities, rooms, and racks

**Fig 4:**
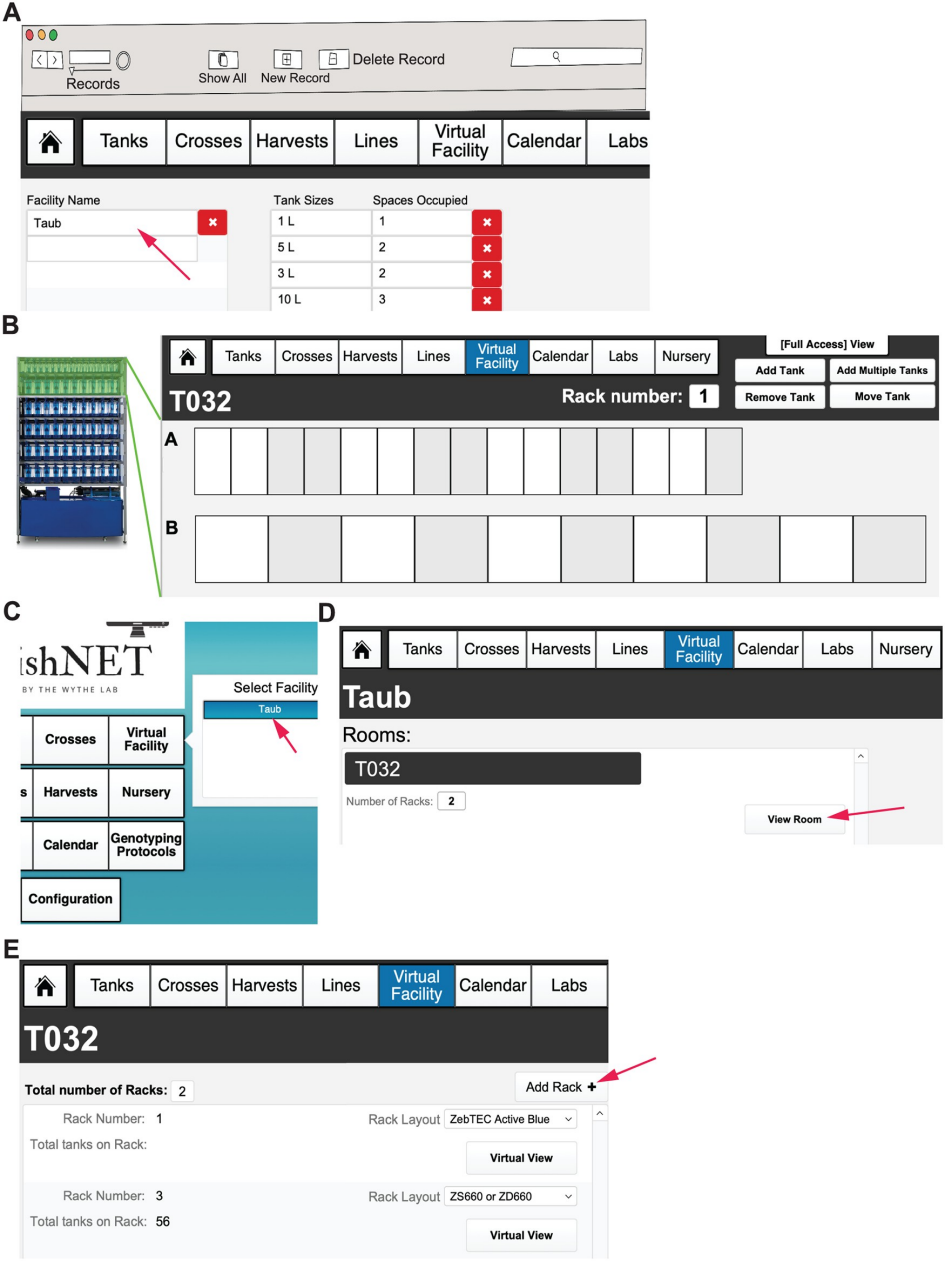
Initial configuration of facility, rooms, and racks. (A) Configuration page where the facility name (indicated with red arrow), tank size, and spaces occupied are initially defined. This page should be set up before using FishNET and should not be modified later. (B) An example of a Tecniplast fish rack (on the left) with a corresponding virtual rack layout (on the right) matching this commercial configuration. (C) After configuration, the Virtual Facilities button will now feature a dropdown list of Facilities to choose from on the landing page (indicated with arrow). (D) Within a facility, multiple separate rooms for housing zebrafish racks can be added by selecting “Add Room.” (E) Inside each room, multiple fish racks can be added to house zebrafish; to add a rack, simply select “Add Rack” (indicated with arrow) and chose the appropriate rack layout according to the manufacturer design.

After generating an administrator profile, the next step is to configure the facility (or facilities). This functionality is restricted to the administrator of the database and not individual users. From the landing page, select the “Configuration” button. In this layout, in the first column, the name of the facility needs to be entered (Fig 4A). In the second column, the different types of tanks being used are specified by the size and number of spaces they occupy on the rack(s). Following the same nomenclature, indicate the different tank capacities (for instance, if you are using a 5-liter tank, enter “5L”) ordered from smallest (nursing tanks) to largest. The “Spaces Occupied” column is used as an approximation of the size of the tank in the zebrafish rack. Fig 4B shows an example set-up of a Tecniplast rack and the corresponding spaces occupied for tanks in each row. This information is used to create a customized virtual rack view. After this initial configuration has been created, the number of rooms within a facility must be specified. This can be done by selecting “Virtual Facilities” from the landing page; upon clicking the button, a list of facilities should be shown in a pop-up scroll-down menu (Fig 4C). Selecting a facility will direct the user to the “Facility View” layout. Here, the option to “Add Rooms” can be found (Fig 4D). Selecting “Virtual Facility” from the main menu bar can also direct the user to the rest of the facilities where rooms need to be set up. It is important to note that in order to use the Virtual Facility function, all Facilities and Rooms need to be properly labeled prior to inputting any records. This cannot be changed later without having to change all records linked to a Room or Facility. Finally, once the Facility and number of Rooms have been specified (and named), the number of racks per room must be entered by selecting the “View Room” button (which takes users to view an individual room) (Fig 4E). When viewing an individual Room, a user can select “Add Rack” to create digital racks that will reflect the physical room.

**Fig 5:**
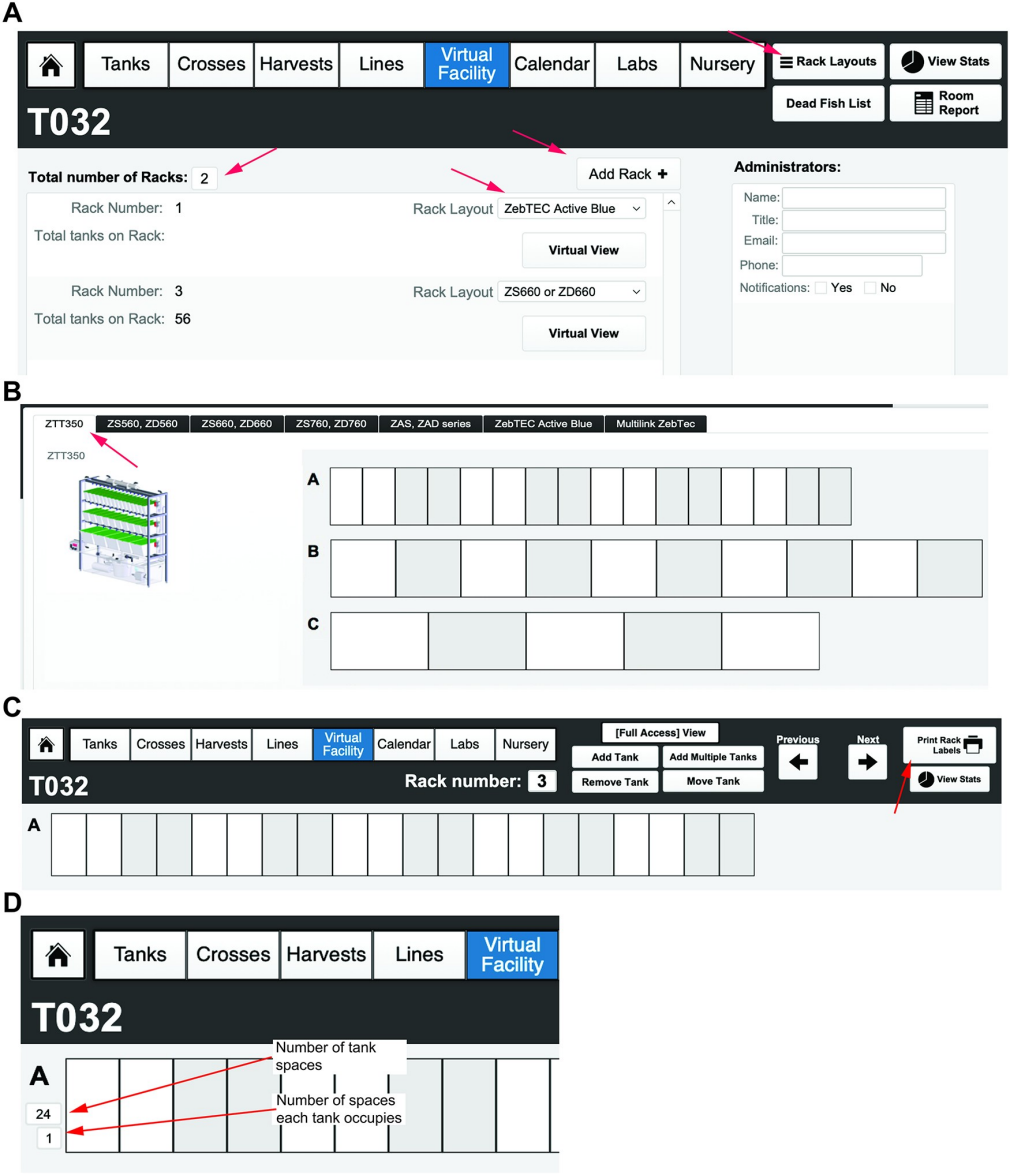
Creation of predefined and custom fish racks. (A) Configuring the number and type of racks within a room of a facility. A dropdown list of predefined fish racks can be found, as well as pictures of each commercially available rack, by selecting the “Rack Layouts” button in the top, right corner of the menu header. Administrators for each room can also be added on the far right, main window. They can receive calendar notifications (fry to transfer to system, turnover of tanks, dead fish, etc.) via email, in addition to users. Racks are added using the “Add Rack” button and chosen from preconfigured options (that match commercially available racks) via the dropdown menu. (B) A picture of a rack and the corresponding virtual rack set-up for one of the predefined rack layouts (using the tabs at the top of the window). (C) Virtual view of the newly set-up empty rack. Each space can be labeled with its own barcode using the “Print Rack Labels” function (indicated with red arrow). (D) Example of a custom rack configuration. In each row, the number of tank spaces and how many spaces each tank occupies can be defined by the user. This can be changed to fit the custom rack specific layout by the facility administrator.

For each rack that is added, the administrator will choose from predefined rack configurations corresponding to the current major commercial aquatic habitat manufacturers (e.g., Techniplast, Aquaneering, and Pentair) (Fig 5A). Pictures of the different aquatic systems can be viewed in the “Rack Layouts” section. Selecting one of these options creates a default layout of tank spaces matching these normal vendor configurations (Fig 5B). Upon selecting a rack layout, a virtual view of the rack will be shown; in the Virtual Facility View, individual positions can be labeled with barcodes by selecting the “Print Rack Labels” function (Fig 5C, indicated with red arrow). If the facility has a unique or custom rack layout, then select the “Custom” option. To set up a custom rack configuration, select the number of tanks in each row (A, B, and so on) by entering in the top number, then specify the number of spaces they occupy by entering in the lower number (Fig 5D). Repeat this for each row on the rack. The “Facility,” “Room,” and “Rack” categories provide spatial organization to the database to create a “Virtual Facility” that can be explored by the administrator, animal husbandry staff, and scientific users alike (more functions within this framework will be discussed after lines and tanks have been introduced). The categories of “Lines” and “Tanks” will be superimposed upon this user-defined framework later (**see Video 2**: https://youtu.be/6aNV2MroMc0).

### Entering laboratories and individual users

**Fig 6:**
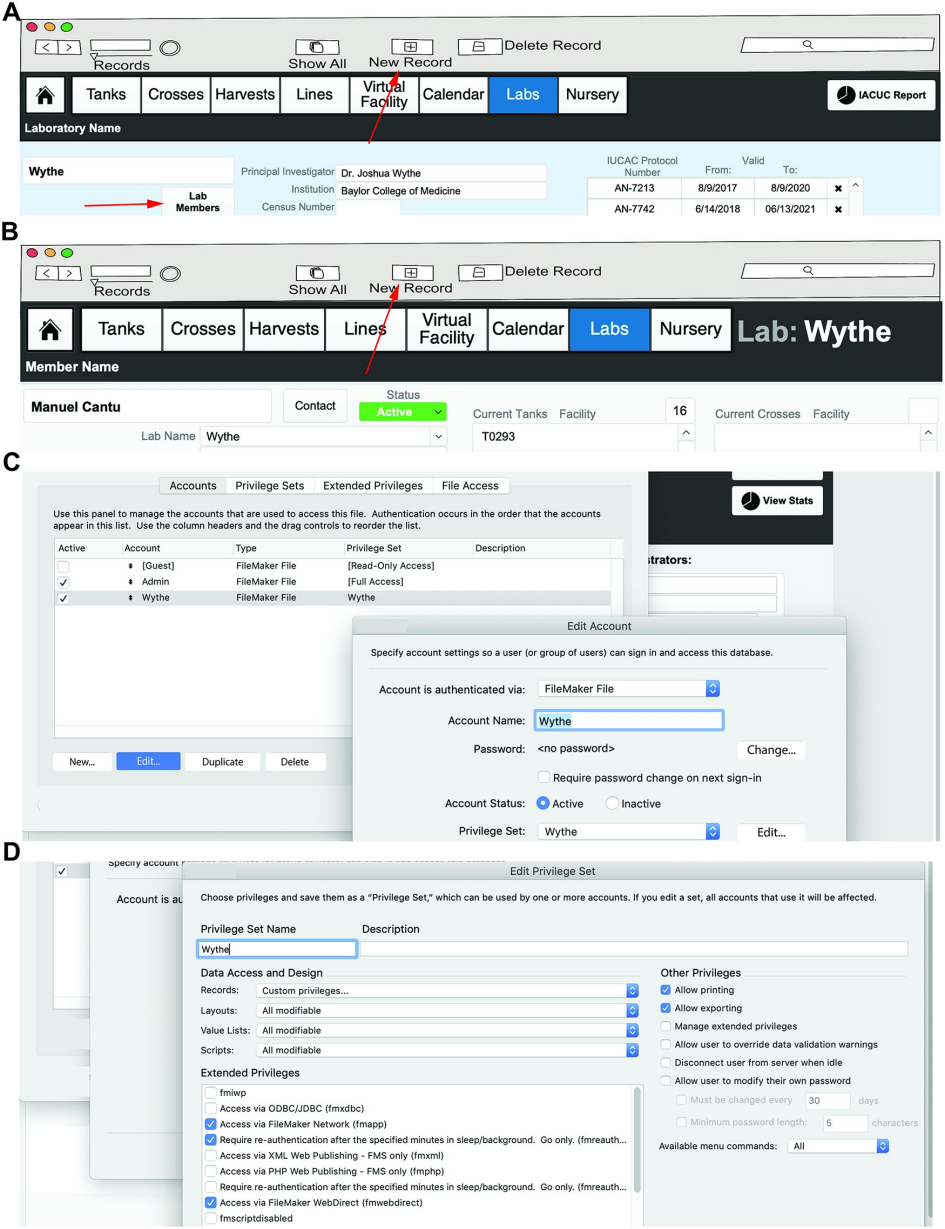
Entering laboratories and individual users. (A) Example of a new laboratory, where the name of the lab, the PI, institution, contact info, and IACUC protocol are all entered. Once lines and tanks have been entered, the lower part of this window will display the number of active tanks, active crosses, and tanks on the nursery for easy reference and billing purposes. Selecting the “Lab Members” button (indicated by the red arrow) will bring you to the next window. New laboratories can be added by selecting “+ New Record” option at the top of the FileMaker menu (indicated by a red arrow). (B) Individual lab user set-up. To add a new lab user, select the “+ New Record” option at the top of the FileMaker menu (indicated by a red arrow). Lab information is automatically populated from the general lab information window, and here individual contact details are entered. Users are defined as “active” or “inactive.” Later, tanks and crosses owned by this individual user will be shown in this window. (C–D) After adding laboratory accounts, an administrator can set up privileges for all users in that lab, as well as other labs, to restrict or permit visualization of lines and handling of fish (e.g., setting up crosses, etc.) by non-lab members. IACUC, Institutional Animal Care and Use Committee.

Next, individual laboratories and users within these laboratories will be entered into the database. Before beginning this section, have the IACUC/animal protocol number(s) for the lab as well as the email address, phone number, and complete name of each user within a laboratory group readily available. The contact information for labs and users will later be assigned to individual tanks on the racks, as well as all crosses and any fry to be raised, allowing for automated reminders about graduating fry to the nursery (or the main system), dead fish notifications, or providing details for contacting other users regarding available lines within the facility. To create a laboratory group, first select the “Labs” button from the landing page (or dropdown layout menu). Once you are in the “Laboratories” layout, click the “+ New Record” button at the top of the FileMaker task bar (Fig 6A). Add all of the pertinent information to this empty field to create a laboratory. Once the lab has been entered, you can then populate it with users by selecting the “Lab Members” button. Within the “Lab Members” layout, click the “New Record” button to add users (Fig 6B). This information will later be used to establish ownership of individual tanks, crosses, and embryos within a facility. Finally, each lab should enter in a valid IACUC protocol number and protocol duration. This information will be associated with all tanks, crosses, and actions of users within a laboratory (and present on all tank, cross, and nursery labels) (**see Video 3**: https://youtu.be/vfBigAefij4).

By default, all new laboratories have access to view and modify the complete database. Below are a series of permissions that an administrator can restrict if they wish to limit individual laboratory members’ access to certain features or functions within FishNET (otherwise, ignore the following steps). Specifically, the following commands will allow individual users to “See and Modify” all information (e.g., genotype, number, sex, location) for tanks owned by all users in a common laboratory, but tanks from other laboratories within the facility (including administrators’ stock tanks) are hidden and the records locked for modification. Administrators, such as animal facility technicians, can view all information and record mortalities and health problems and can work with any tank (e.g., to graduate fry or to retire a sick tank). For this type of access, first create a new user or group account (Laboratory) with restricted access to the database by selecting File/Manage/Security. Then, select “New Account” and make sure the option to authenticate via FileMaker File is confirmed (Fig 6C) (note that the “Account Name” must match the name of the laboratory within FishNet). Type a password for the new account, then select “Privilege Set” and choose “New Privilege Set.” A pop-up window will open, and within that window, change the privilege set name to the name of the laboratory. After the name is changed, make sure all the data access and design options are set to “All Modifiable,” then click Records and select “Custom Privileges” (Fig 6D). A new window will be opened where you can see all of the tables that store information in the database. Each table will have a different setting. Here, you can select “Fish Lines,” then change the “Delete” option to “No.” Select “Tanks” and change the “View” option to “limited,” then enter the following text into the pop-up window: Laboratories::Lab Name = "Wythe", replacing “Wythe” with the desired laboratory. Repeat this same operation for the “Delete” option. In order to complete the set-up for each new laboratory, the administrator must set up the appropriate permissions for all data tables. For a fully functioning lab, refer to Fig 6D and modify each field as shown. Follow the previous instructions for every instance of “limited” privileges, referring to Table 6 for necessary settings (**see Video 4**: https://youtu.be/NrI7TOiSCvs).

**Table 6:** Setting up lab privileges.

Table Name	Limited Fields	Calculation
Tanks	View	Laboratories::Lab Name = “Lab Name”
	Delete	Laboratories::Lab Name = “Lab Name”
Fish Crosses	View	Laboratories Crosses::Lab Name = “Lab Name”
	Delete	Laboratories Crosses::Lab Name = “Lab Name”
Harvest Assets	View	Laboratories Harvest::Lab Name = "Lab Name"
	Delete	Laboratories Harvest::Lab Name = "Lab Name"
Harvests	View	Laboratories Harvest::Lab Name = "Lab Name"
	Delete	Laboratories Harvest::Lab Name = "Lab Name"
Lab Members	View	Lab Name = "Lab Name”
	Delete	Lab Name = "Lab Name”
Laboratories	View	Lab Name = "Lab Name”
Dead Fish	View	Laboratories::Lab Name = "Lab Name"
Event	View	Lab = "Lab Name"
	Delete	Lab = "Lab Name"
Nursery	View	Laboratories Crosses::Lab Name = "Lab Name"
	Delete	Laboratories Crosses::Lab Name = "Lab Name"

### Entering lines

**Fig 7:**
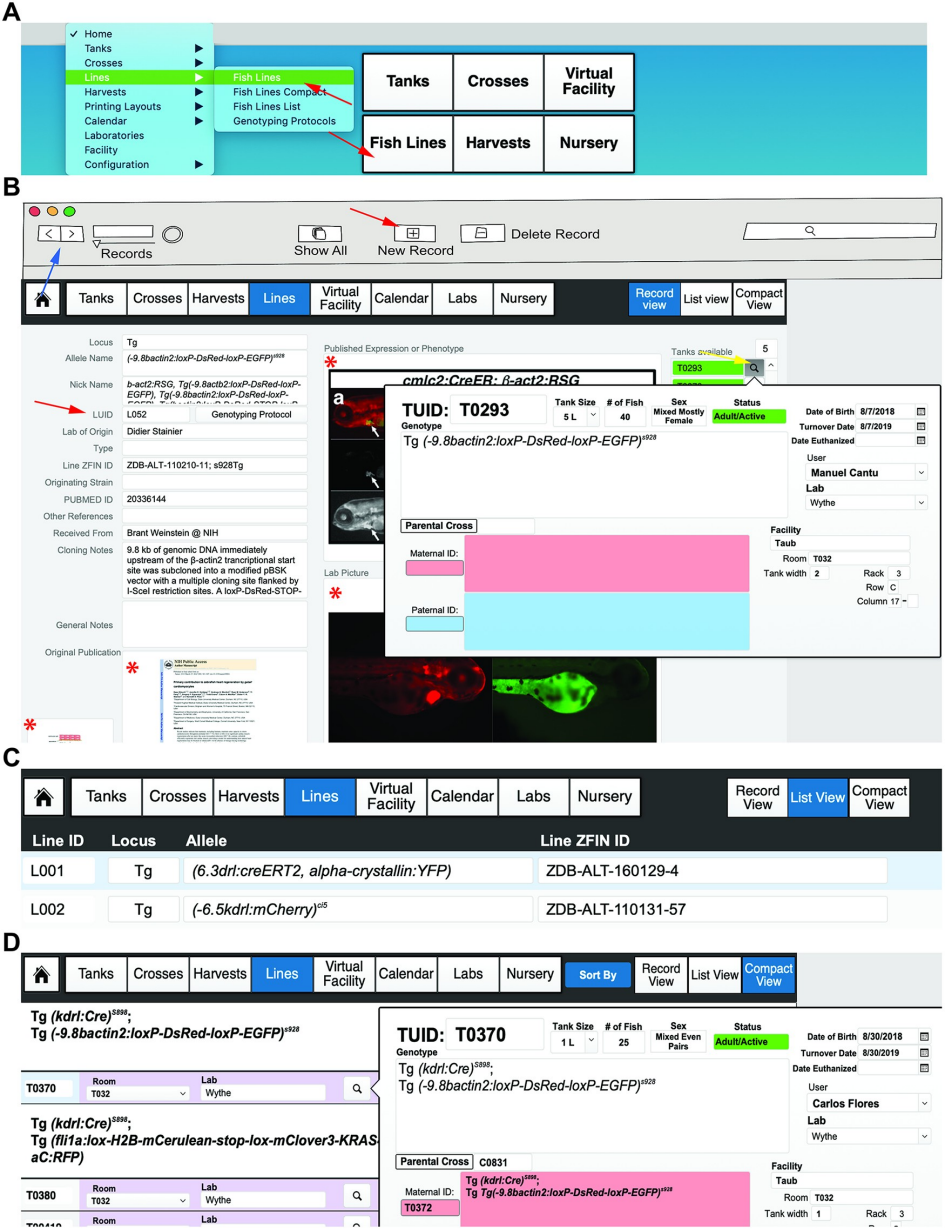
Entering lines. (A) The “Fish Lines” layout can be accessed from main layout dropdown menu, or by clicking “Fish Lines” on the main landing page (each indicated by the red arrows). (B) A new line information page can be created by selecting “New Record” (indicated by the red arrow in the header). Users can move through all records using the forward and backward arrows (top left of the header, indicated by the blue arrow). Each new record is assigned an LUID, indicated by the red arrow. This is an example of a fully annotated line record. Containers where external pictures or file attachments can be placed are indicated with an asterisk. Any tanks available for a given line will be displayed on the far-right side of the main window under the “Tanks Available” label. The tank labels are color-coded according to their age and mating status. Selecting the magnifying glass will bring up a pop-up window with a detailed overview of an individual tank (indicated by yellow arrow). Lines can also be browsed in list view (C) or compact view (D) for easy navigation, with more detailed information (TUID, tank size, number of fish, date of birth, etc.) easily available by clicking the magnifying glass icon. LUID, line unique identifier; TUID, tank unique identifier.

Lines, in this case, refers to distinct alleles that follow nomenclature established by the zebrafish information network (ZFIN) (https://wiki.zfin.org/display/general/ZFIN+Zebrafish+Nomenclature+Conventions). Novel lines, such as those generated by Clustered Regularly Interspaced Short Palindromic Repeats/CRISPR-associated protein-9 nuclease (CRISPER/Cas9) mutagenesis, can be also be entered in FishNET. To enter an allele, from the landing page, select the “Lines” tab (or select the “Fish Lines” layout from the Layout pulldown menu) (Fig 7A). If your database has no lines entered, you will be taken to a blank record. Under “Record View,” you will only be able to view one line or record (e.g., a unique allele) at a time. Use the forward and backward icons at the top upper left of the database window to scroll through the records manually (Fig 7B, indicated with blue arrow).

To create an allele in “Record View,” populate the following fields in the blank record: locus, allele name, nickname, line unique identifier (LUID), lab of origin, line ZFIN ID, originating strain, PUBMED ID, laboratory the line was received from, cloning notes, and general notes (Fig 7B). Importantly, whatever designation is used for the “Allele Name” field will be redeployed throughout the database in dropdown menu choices, ensuring uniform nomenclature within a research groups, across other labs, and throughout entire facility (**see Video 5**: https://youtu.be/ONgTj538AmU). Each line that is entered is assigned an LUID number (beginning with L). Each subsequent record is automatically assigned the next available LUID number (L + 1) (Fig 7B). FishNET also allows for users to upload and store attachments associated with each line, such as associated publications, representative images of example phenotypes, or expression patterns that can function as a reference for sorting offspring (Fig 7B, indicated by asterisks). Additionally, one can link genotyping protocols to individual lines such that the protocol can be accessed by selecting the “Genotyping Protocols” layout or button within the “Record View” field (these will be discussed in more detail in the following section).

While users or owners of the line are not defined when a line is created, later, if tanks are populated with a line and placed on a rack, they will be visible in the “Lines: Record View” window under the “Tanks Available” heading. Only live tanks will appear within this window. Clicking on the magnifying glass provides a detailed view of the tank, including its genotype, date of birth, sex, number, the owner of the line, its status (e.g., juvenile, adult), location in the facility, and the paternal and maternal animals that generated the tank (Fig 7B).

To view all lines that have been entered into a database, within the “Lines” layout, select “List View” (rather than the individual “Record View”) (Fig 7C). This generates a simple, scrollable list of every allele, including the locus, allele, and ZFIN ID. To define a new locus in the “List View” layout, simply input text into the space provided (or select “New Record”), and the new locus will be indexed and will appear as an option in any subsequent input. Enter the correct genetic manipulation, locus, allele, and ZFIN information, and the rest of the database will populate the allele name using this designation. For adding additional details, select the “Record View” layout and finish entering in all pertinent information.

To view all lines that are actually available within a facility or user group, select “Compact View.” Users can navigate this view using the “Sort By” button to segregate the animals by any of the following criteria: genotype, facility, lab, or user. This view will show users the TUID, facility, and lab that own any tank(s) of a given genotype. Additionally, users can click on the magnifying glass for a detailed view of that particular tank (location, parents, age, etc.) (Fig 7D).

### Entering genotyping protocols

**Fig 8:**
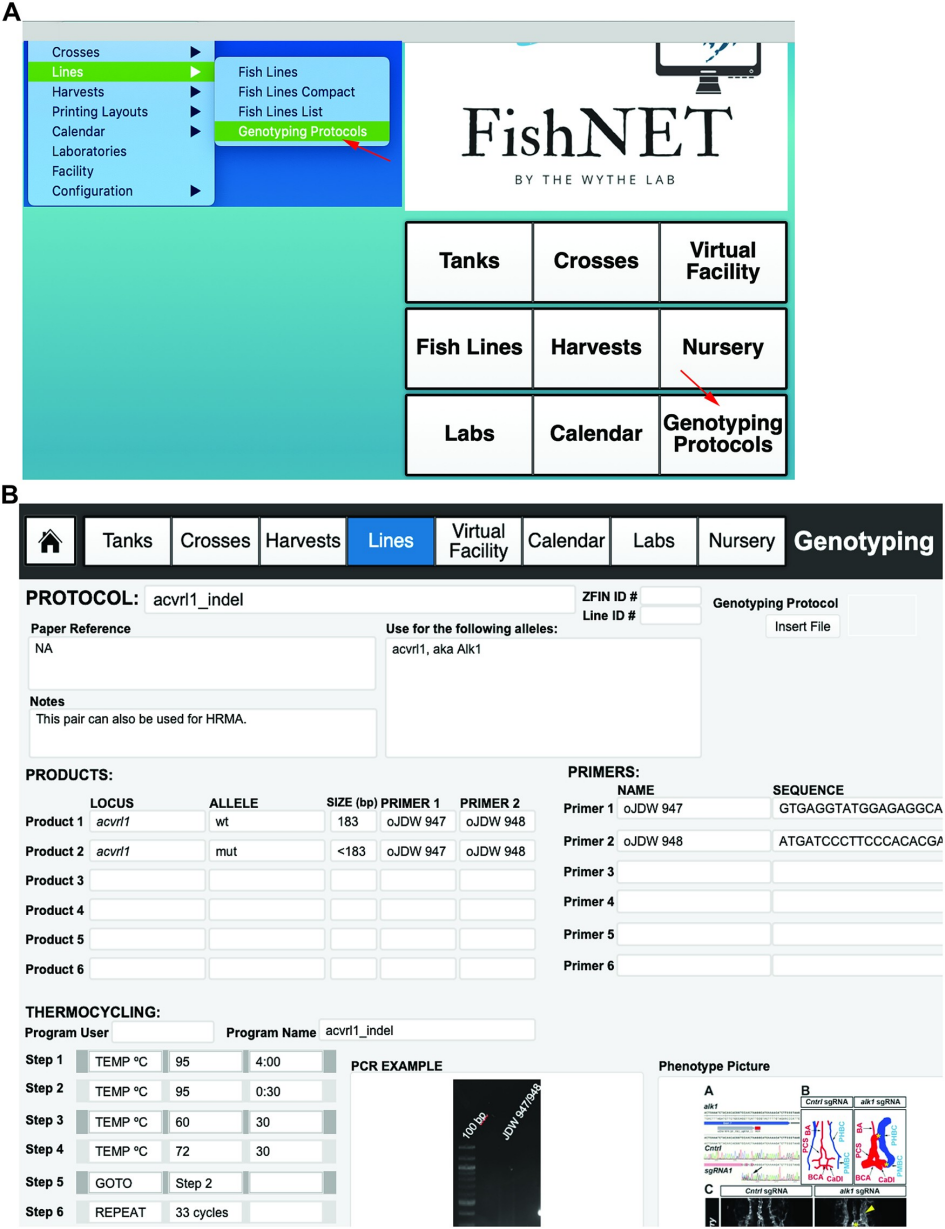
Entering genotyping protocols. (A) Genotyping protocols can be accessed from the landing page via the “Genotyping Protocols” button or via the dropdown menu under “Lines” and “Genotyping Protocols.” (B) An example genotyping protocol, where primers and PCR conditions and images, as well as phenotypic information, can be imported.

To create a genotyping protocol, from the landing page, select “Genotyping Protocols” (Fig 8A, indicated with red arrow), then select the “New Record” button. Here, you will name the protocol and enter the ZFIN identification number and any notes, as well as PCR primers and thermocycler conditions, expected product size, and a representative agarose gel image, by dragging and dropping any .PNG or .JPEG file into the PCR example container (Fig 8B). For optimal viewing, gel images should be resized to 4 inches width and a resolution of 300 pixels/inch. To scroll through available protocols, use the FileMaker forward and backward keys available at the top of the screen or use the search window.

### Creating, locating, moving, removing, and retiring tanks

**Fig 9:**
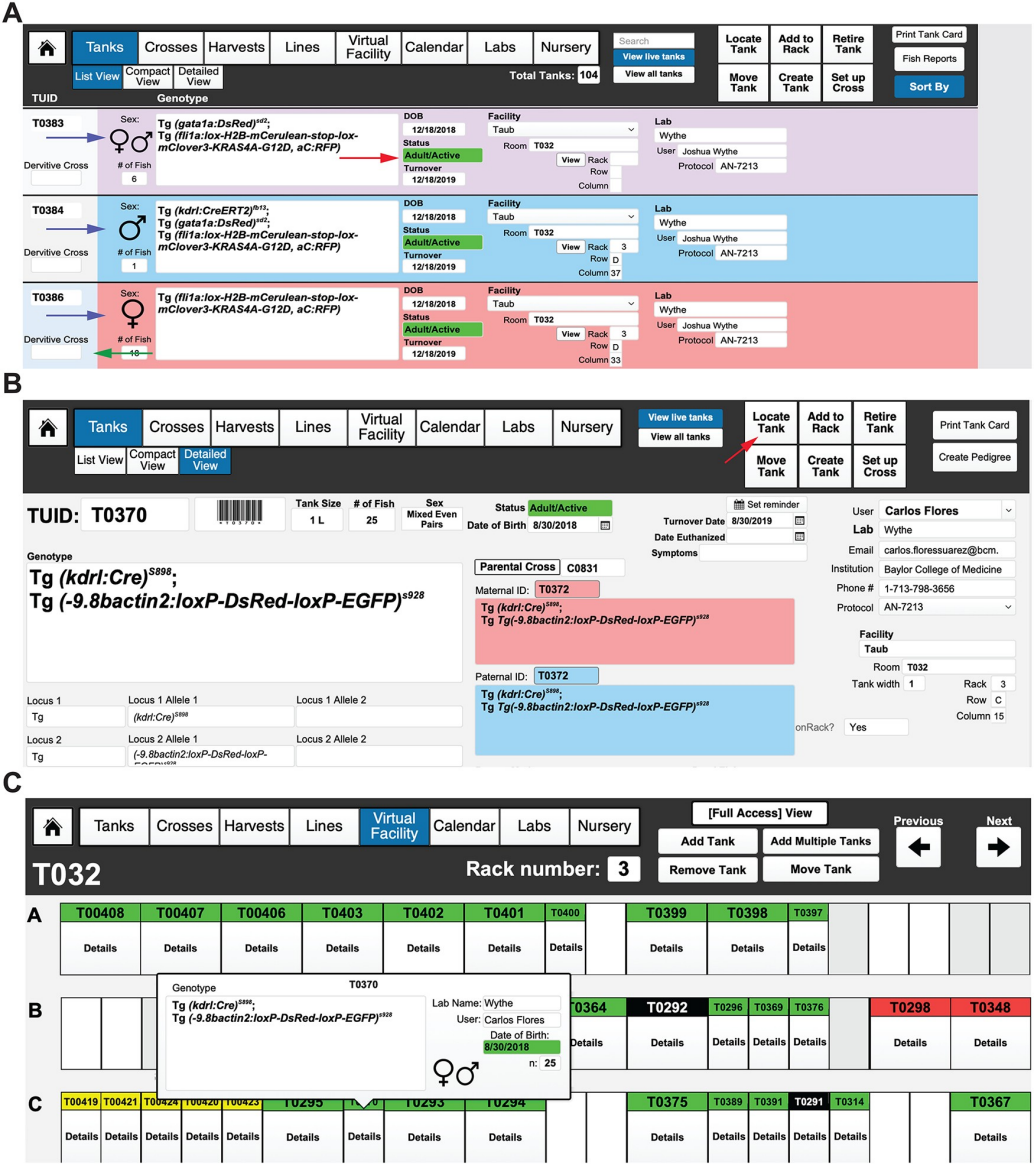
Entering and viewing tanks. (A) “List view” of all “Live” (active) tanks, in this case sorted by “Facility.” All active tabs for viewing are indicated by blue coloring. The age and status of each tank is indicated by color (see red arrow), while the sex of each tank is indicated by the background color (blue for male, pink for female, and purple/pink for mixed sex tanks, indicated with blue arrows). Selected tank is indicated by a bar changed to light blue (indicated by green arrow). (B) Detailed view of a tank with the derivative cross (C0831) and genotype/TUID of the parents, date of birth, date for turnover, and user/owner of the tank all indicated, as well as the facility and position. To view the virtual position of the tank, select “Locate Tank” (indicated by the red arrow) in the “Tanks Detailed View” layout, which will bring you to a new window. (C) The Room will be indicated on the upper left of the header menu (in this case, T032), while the Rack will be indicated in the center of the header menu (in this case, rack 3). The position of the tank of interest will be indicated by a blue label. In this case, TUID 0370 is in Room T032, Rack 3, Row C, position 15. Clicking on “Details” will show the genotype, sex, age, and number of fish in the tank, as well as the owner of the tank. All other tanks are colored according to their age and mating status (white ≤ 2 weeks old, yellow ≤ 3 months old/juvenile, green ≥ 3 months old/active, and red ≥ 1 year old). TUID, tank unique identifier.

The underlying logic of FishNET that enables the tracking and searchable features relies upon a TUID that is assigned to every new tank. From the “Tank List” layout (Tanks > List View or Landing Page > Tanks), one can see a list of all tanks, each with a TUID, stored within the database (Fig 9A). All pertinent information (sex, genotype, age, owner, and location) are present in this view. To create a tank, click the “Detailed View” tab (or, from the main dropdown menu, select “Tanks” and go to “Tanks Detailed View”). In that layout, select “New Record.” Then, select the loci and the appropriate allele from the dropdown list (that should have been populated by following the instructions outlined in Entering Lines). After this, select the “User” of the tank (this will autopopulate the user’s lab, email address, institution, phone number, and IACUC information). If the TUID of the maternal or paternal parent tank is known, enter it now to establish a pedigree for this new tank. The TUIDs can be entered manually, or you can select the field(s) with your cursor and then scan a barcode for the animal of interest. Be sure to enter the date of birth, tank size, and number of fish for the TUID record. The status of the line will be color-coded according to the date of birth (fish younger than 2 weeks are white, fish older than 2 weeks but less than 3 months are yellow, fish older than 3 months but younger than 1 year are green, fish older than 1 year are red, and retired or euthanized tanks are black). Additionally, the database automatically creates a turnover date for 1 year after the date of birth. Users can choose to receive an automated email reminder for line turnover by selecting the “Set Reminder” calendar button above the “Turnover Date” field (but this must be done after establishing the User/Owner of the tank to set up an email address for the reminder). Finally, a user must assign a location for the tank by selecting the “Facility” dropdown menu and entering in the Rack, Row, and Column for the new tank. After entering that information, select the “Add to Rack” button to place the tank in the virtual facility. Alternatively, one can click the “Add to Rack” button in the header field and then scan in the barcode location on the rack followed by the barcode on the tank to establish the location of the tank (**see Video 6**: https://youtu.be/fp1ZA1NSeEI). Once the user adds the tank to a rack, selecting the “Locate Tank” button will show where the tank in question is located within the virtual rack window (Fig 9B).

### Setting up crosses

**Fig 10:**
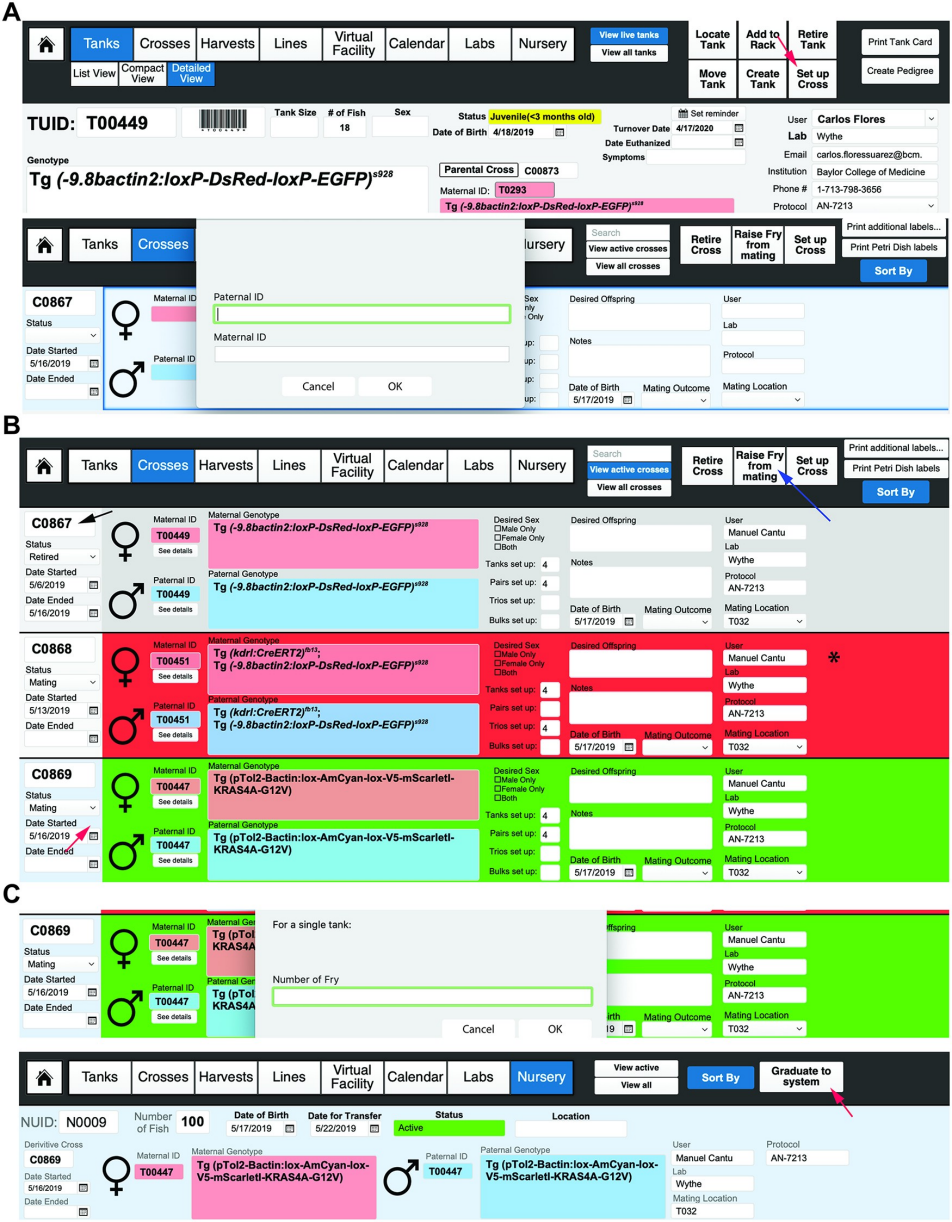
Setting up crosses. (A) Crosses can be set up from the “Detailed View” by selecting “Set Up Cross” button (far-left screen image), indicated by the red arrow, which will take you to a new screen (far-right screen image) where you can either manually or by barcode reader enter the TUID for the dam and the sire for the cross. Selecting the “Set Up Cross” function automatically generates a new CUID number and record for tracking purposes. The crossing function also allows you to record the success of the mating for tracking the fecundity of a tank (and overall mating at the level of a rack, room, or facility) (not shown). (B) The “Crosses” layout displays either “Active” or “All” crosses. The far-left column (indicated by the black arrow) displays the CUID of a cross at the top, with the status of the cross (either mating or retired) indicated below in the dropdown menu. Below that are the dates the mating was started, as well as the date the mating was ended. The blue background in the far-left column (indicated by the far left, red arrow) shows users which cross is currently selected (compare the blue background indicated by the red arrow in C0869 to the light gray background in the far left of C0867 for reference). Each cross is also color-coded for easy visual reference (indicated by the asterisk on the far-right side). Green = an active cross; red = an active cross, but more than 2 days have passed since it was set up; and gray = retired crosses. After a cross has been ended, in the top right corner, a user then selects to retire a cross (indicated by blue arrow), after which the background color on of a cross will automatically change to gray (compare the background color of C0867 between the left and the right images). The upper right header menu also has options to raise fry from the mating (indicated by blue arrow), to set up a new cross, to print more cross labels, or to print labels for petri dishes for embryos. (C) Selecting “Raise Fry from Mating” will ask the user for the number of embryos that will be kept in each tank/petri dish for raising. After selecting “OK,” a second window will ask if there are additional embryos to raise from the same cross (not shown). Once the user indicates that no more fry will be raised, a new NUID record (bottom image) will be created. Here, the NUID record, as well as the identity of the sire and dam, number of fish, and other details will be autopopulated from the CUID record. Once the fry are ready to move onto the main system, the user can select “Graduate to system” (indicated by the red arrow in the far-right image). This function creates a new TUID autopopulated with all of the pertinent NUID and CUID information (not shown). CUID, cross unique identifier; NUID, nursery unique identifier; TUID, tank unique identifier.

Each mating generates a CUID number to track every mating and to generate pedigrees. To create a cross, select the “Crosses” button from the header or landing page menu (or select “Cross” from the pulldown menu). Then, in the header menu, select “Set Up Cross.” This option can also be found in the Tank Detailed View (Fig 10A, top). A pop-up window will appear asking for the paternal TUID and the maternal TUID (Fig 10A, bottom). These can be entered either manually by text or by reading the barcode of each parent tank. Next, a pop-up window will ask what type of mating is being setting up: a bulk cross, a trio mating (defined as one male crossed to two females), or a pair. The next window will ask how many of this type of mating are being set up (e.g., 8 pairs). After filling in this field, the application will show you a mating label while asking you if you want to repeat the cross. If you are only setting up one type of cross (e.g., just pairs) and selected the appropriate number, then chose “No.” If you wish to set up another type of mating from this cross, such as a few trio tanks, then select “Yes.” The cycle repeats until you chose to no longer repeat this cross. After choosing “No,” the screen will return you to the “Crosses” layout. To simplify the view, you can choose to “View Active Crosses” rather than “View All Crosses.” In the header field, you will now have the option to print mating labels for each of the individual mating tanks that you set up. These labels display the genotype, sex, TUID, and location of the parent tanks, as well as the owner of the cross. If additional mating labels are needed, select the “Print Additional Labels” field in the header. We also use these to label 10-cm plates of embryos from crosses (and thus print extra labels). Active crosses that are less than 1 day old will be labeled green in the “Crosses” layout, while active crosses more than 1 day old will be labeled red to indicate that the fish should be returned to the system (or fed). Retired crosses will be colored gray (and users should retire every cross once the mating pairs are taken down and returned to the system). This convenient color-coding system enables easy visual tracking of active crosses within a facility or user group (Fig 10B) (**see Video 7**: https://youtu.be/1EkTnV2AfdI).

### Raising offspring/graduating fish and nursery

FishNET also has the capacity to track all larvae that are destined to be graduated to the main system, enabling real-time tracking of all tanks on a nursery, as well as the success of fish survival rates, through the “Statistics” button within the “Virtual Facility” layout. To raise fry from a cross, select the “Raise Fry from Mating” button in the header menu (Fig 10B). A pop-up window will ask you for the date of birth for the fry, as well as the number of fry for a single tank to raise (this will help determine survival on the system, as discussed later). Within the “Nursery” layout, each tank of fry is assigned an NUID. After filling in this information, a new pop-up window will ask if you wish to raise more fry from this cross (e.g., additional plates/tanks).

**Fig 11:**
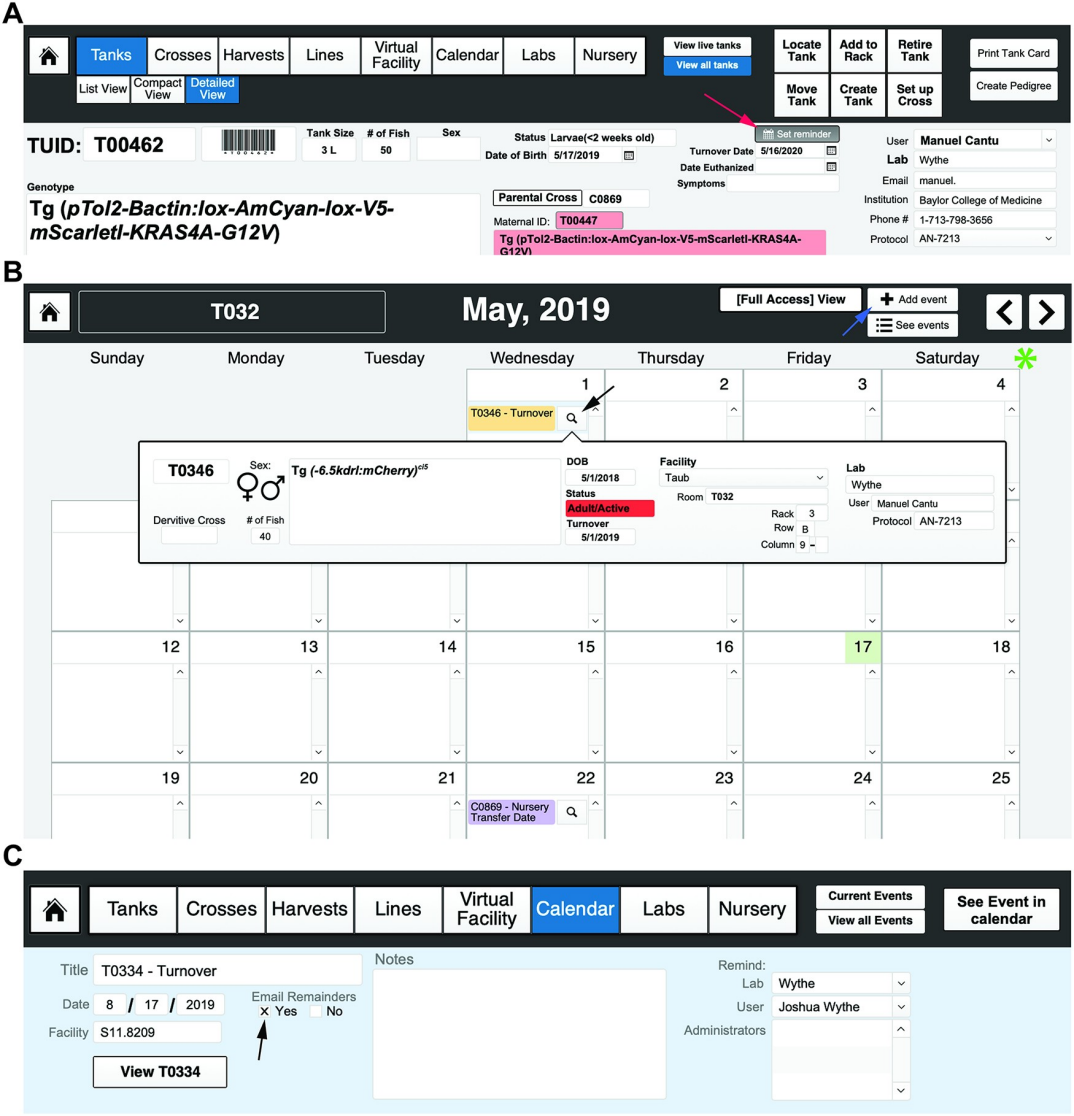
Setting up calendar reminders. (F) Once a new TUID is created (after graduating fish from nursery), a user can automatically set a reminder to turn the line over 1 year from the date of birth by selecting the “Set Reminder” button (indicated by the red arrow in the far-left image). The middle image shows the “Calendar” layout (in this case for the month of May, 2019 for room T032). More “Events” can be added (using the button indicated by the blue arrow). Clicking on the magnifying glass (denoted by the black arrow) will create up a pop-up window showing the details of an event. Months can be changed using the forward and backward buttons on the far right of the top header menu (indicated by the green asterisk). (C) Detailed “Event” layout, where FishNET has the capacity to email a reminder to the lab user and/or room administrator for any given event (using the contact details supplied in the laboratory set up). TUID, tank unique identifier.

At day 5, select the “Graduate to System” button in the top right of the header. Upon selecting this button (located in the header field of the “Nursery” layout), a pop-up window will ask the user to enter NUID(s) that will be used to form a new Tank (in the event that more than one dish of fry of the same genotype are used to create a new tank on the system) (Fig 10C, **right**). This automatically generates a new, unique TUID record that contains all of the previous NUID information (allele, owner, strain, etc.) (Fig 11A). Once a new TUID is created, a user may select the “Add to Rack” function, as described previously, to “place” the tank in the virtual main system (**see Video 8**: https://youtu.be/9wATt56mVsg).

### Calendar

The calendar layout/function (Fig 11B) enables users to access a facility-wide calendar with all related events (crosses set up; system reminders such as change baffle size, alternate food, graduate to main system; etc.). Selecting “Add Event” will take the user to a field where the title of the event, date, and facility are entered. Finally, the calendar function has an option for email reminders that automatically populates information using the laboratory user field(s). This function can also be used to copy administrators or animal husbandry staff on reminders.

### Harvests

**Fig 12:**
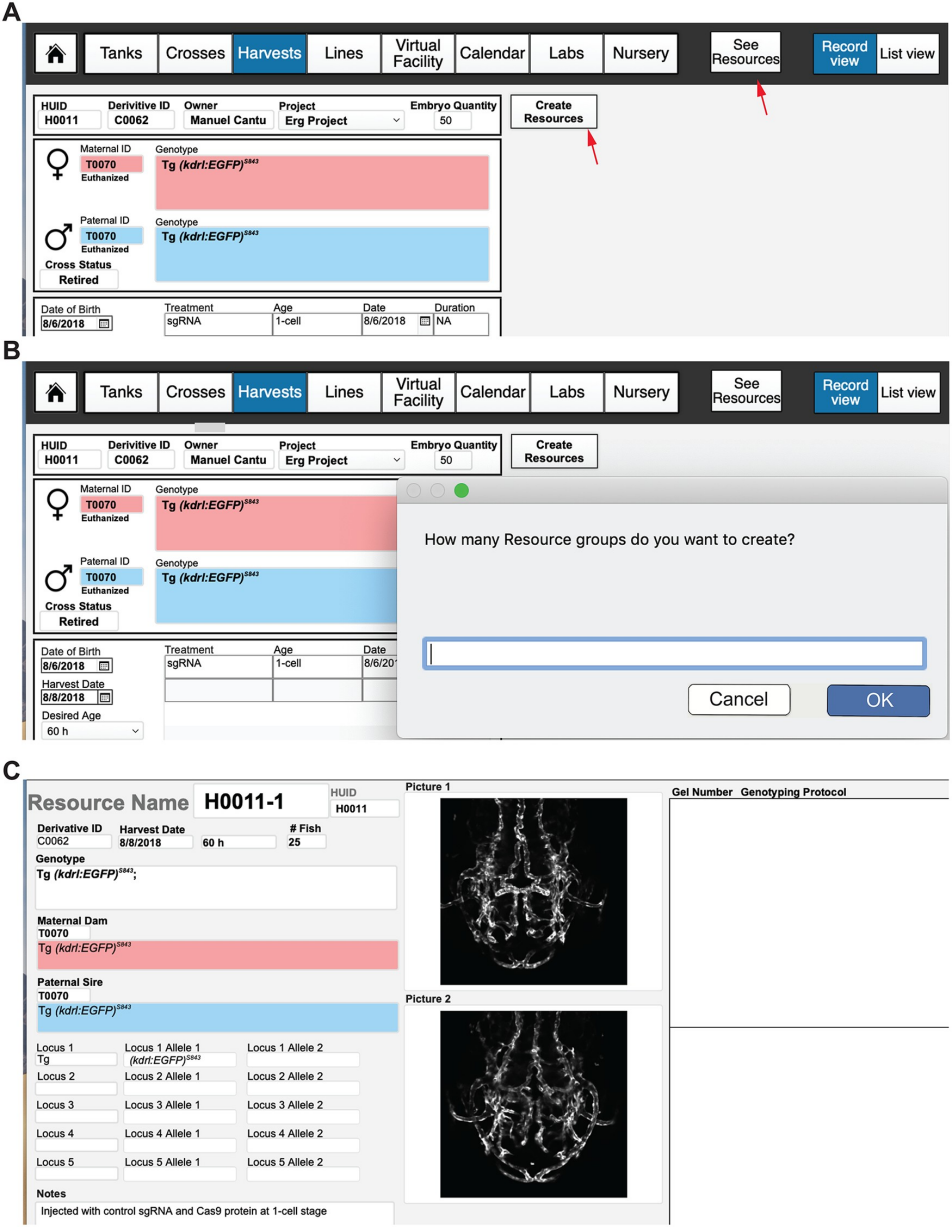
Using FishNET to track harvest records. (A) Embryo harvest template. Creating a new record in the “Harvests” layout will create a new HUID record, where information such as the cross used to produce embryos, the genotypes and TUIDs of the parents, the date of collection, and number of embryos can be entered. Information regarding any chemical or genetic treatments, the age of collection, any genotyping to perform, and downstream tissue processing can be entered into this record as well. (B) After an embryo/adult quantity is added, a user can select the “Create Resources” tab to create a unique Harvest ID (H000X-1 if one resource, 1 and 2 if two, and so on) for each embryo group or adult fish that is processed. Selecting “Create Resources” will prompt the user to select the number of groups that embryos or adults are going to be divided into. If the original embryo quantity is 50, the user could make 2 groups of 25 embryos, each with a different experimental condition. (C) Viewing the resources takes the user to an individual record view of all “resources” associated with that harvest. Information regarding genotype, tissue processing and images of the embryos can store here to create permanent records of any experimental harvest. HUID, harvest unique identifier; TUID, tank unique identifier.

Another novel, to our knowledge, feature of FishNET is the ability to track the pedigree of embryos or adults that are used for experiments (as well as all experimental conditions) in the “Harvests” layout. This layout generates a novel HUID that pulls from either the parental cross CUID (for embryos) or the TUID (for an adult) to create a unique record with the date of the harvest, experimental treatment, and number of fish collected (Fig 12A). Additionally, using the “Create Resources” tab, one can generate detailed records for every embryo or adult within a harvest (Fig 12B). While nonessential for facility management, many individual labs may find this functionality helpful for tracking embryos or adults that have been harvested and stored in the freezer and for keeping track of sections, mRNA, or protein samples. Tagging samples with a unique Resource ID and labeling a sample “H0001-1” is easier and clearer than writing out an entire genotype, sex, date, and treatment on one slide or an Eppendorf tube. Furthermore, there will be a permanent record of the parents, animal, genotype, and treatment associated with this HUID. Additionally, images (such as genotyping gels) and other relevant data can also be pasted within these “resource” pages (Fig 12C).

### Automated genotype annotation for harvest

To automate annotation of genotyping gels, we created an Apple script (FishNET_Genotype.scpt file) that can take the record list from the harvest section of FMPA into Adobe Photoshop Creative Cloud 2017 to annotate images of PCR gels (this function is only available in Mac OS X). This requires a completed genotyping protocol (S1A Fig), which can be found under Lines/Genotyping Protocols. A special security permission is also needed to allow Apple scripts in FMPA: select File/Manage/Security… Select Extended Privileges and give full access to “fmextscriptaccess.” To start, download the script (S2 File). Open the script in Script Editor and follow the simple instructions to adapt it to your computer (S1B Fig). We recommended adding a shortcut for the script to your menu bar. From the script button on the menu bar, select “Open Scripts Folder/Open Users Script Folder” and deposit the downloaded script file there (now the script can be run from the Script menu in the menu bar). Before running the script, a genotyping gel should be open and resized in Photoshop to 4 inches width by 3 inches wide, with a resolution of 300 pixels/inch (S1C Fig). Run the script and enter the HUID number you want to annotate. Make sure all names match the correct bands on the image, then select “OK” in the pop-up window asking, “Are ready to add the gel to the database?” Select “Yes” to create a new genotyping record. Drop the new annotated gel image into the empty field and select the appropriate name of the genotyping protocol as seen in S1D Fig. The annotated image is now present in the detailed view of all records linked to the selected harvest ID (S1E Fig).

### Importing existing data to FishNET from Excel or FileMaker

**Fig 13:**
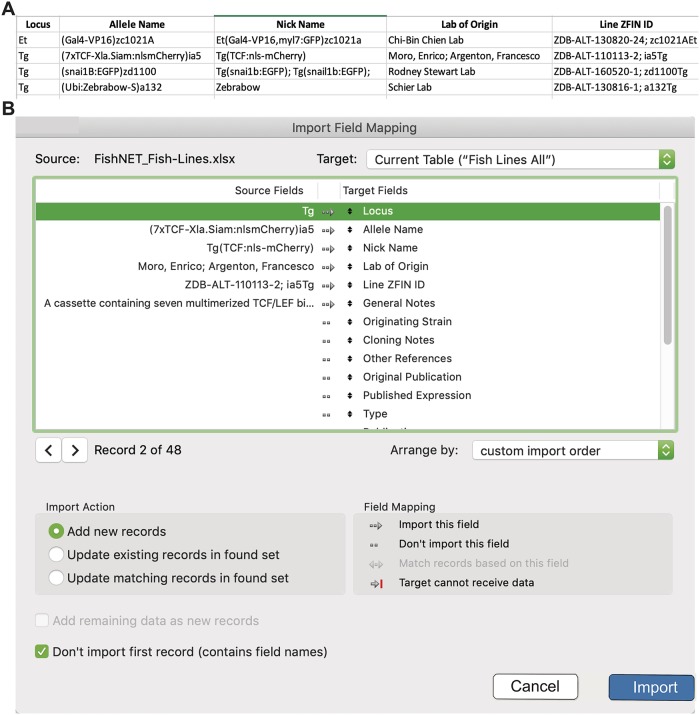
Importing data into FishNET. (A) Screenshot of the Excel template used to import lines into FishNET. All fields should be filled accordingly. (B) Import Field Mapping showing the correct configuration to import new lines into FishNET.

Because many laboratory groups use Excel spreadsheets to manage their existing colony, we provide a simple way to import this information into FishNET. Once the laboratory, members, facility, and rooms are set up in FishNET, one can import preexisting line data. In Excel, ensure that the following columns are populated: Locus (Tg, Et, etc), Allele Name, Lab of Origin, and General Notes (Fig 13A and S3 File). After all fields are entered, save the document in Tab-delimited text (txt) format. To import these data into FileMaker, go to the “Lines” tab and select File/Import Records/File. Next, select the .txt file where the data were saved. Once selected, the user has to match the source field from Excel to the target fields in FileMaker Pro. Activate field mapping and importing by selecting all fields that should be imported (the middle symbol between “Source Fields” and “Target Fields” should change to an arrow) (Fig 13B). “Add new records” should be selected, as well as “Don’t import first record (contains field names)” (Fig 13B). Similarly, it is possible to import data from a preexisting FileMaker database. In the original database, go to the layout you wish to export (Tank list, Lines, etc.) and select File/Export Records. Then, “save” the new file as an FMPA-type (fmp12) file. Next, select the fields to be exported and select “Export.” Finally, to import these data into FishNet, follow the above instructions, selecting the new .fmp12 file as the source of import.

### Reporting capabilities

**Fig 14:**
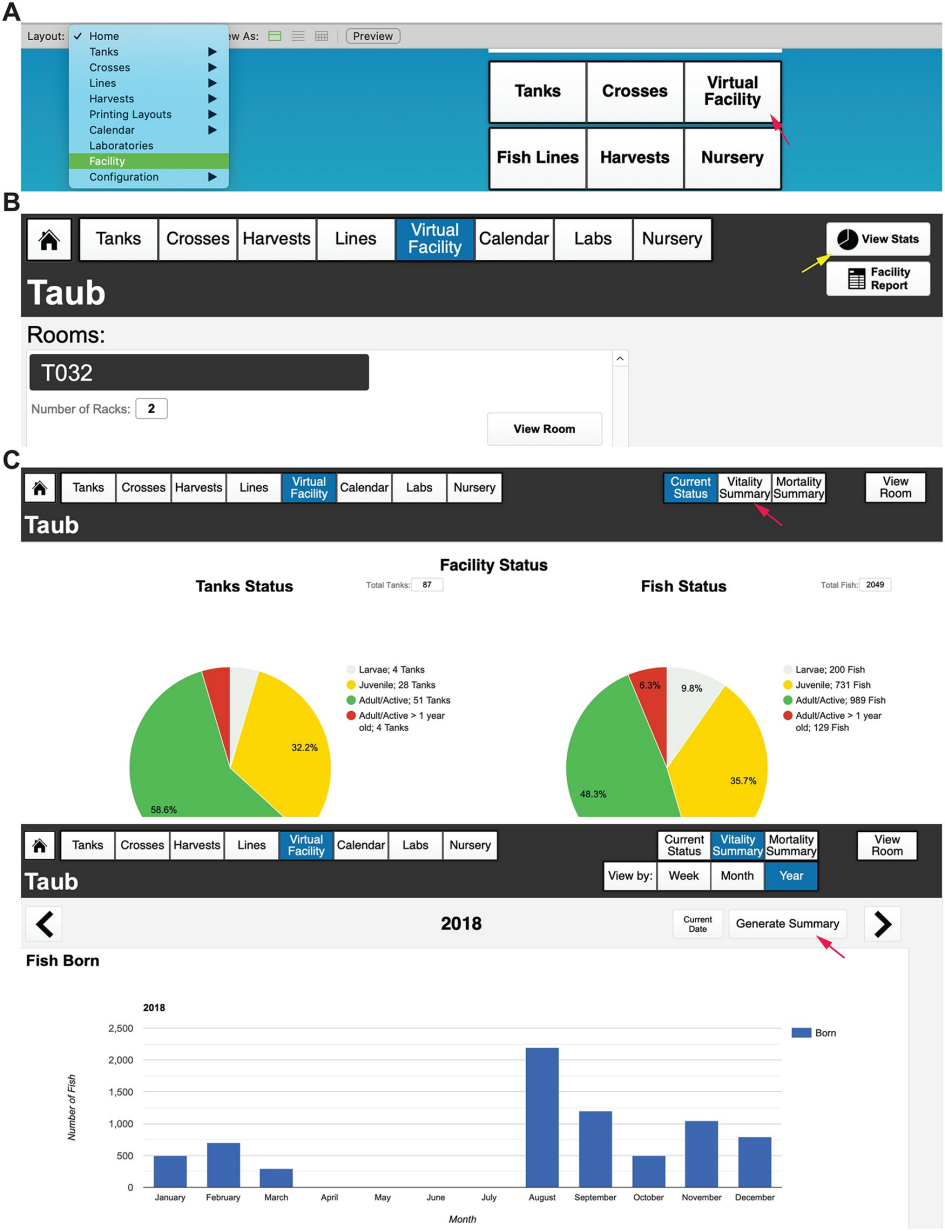
Generating fish statistics with FishNET. (A) Navigate to the “Virtual Facility” from either the landing page (left) or the header tabs. (B) From the “Facility” page, selecting “View Stats” (yellow arrows) will take the user to a statistical report for either the facility, room, or individual rack. (C) shows the “View Stats” layout for an entire facility, with the “Current Status” of all tanks in that facility on the left and all individual fish on the right. From here, one can also view the “Vitality Summary” (indicated by the red arrow in the header menu) for the entire facility, room, or rack. “Generate Summary” (indicated by the red arrow) in the “Vitality Facility Summary” layout creates a report either for the past year, month, or week displaying all recorded fish born (embryos) from crosses, dead fish found in the facility, and fish euthanized for a comprehensive view of the vitality of a facility or room.

We placed an emphasis on the reporting capabilities of the database, which allow for storing fish census and usage reports for the whole facility in an exportable, Excel-compatible format file. This graphical report provides an overview of all fish morbidities and mortalities, and individual fecundity records are maintained for each line across the entire facility. FishNET also tracks all animals, can generate user-defined fish use reports (as required for annual IACUC protocol reporting), and, as previously mentioned, generates graphical reports for water quality data. Within the “Virtual Facility” layout (Fig 14A), selecting the “View Stats” function (Fig 14B) will take users to a graphical report of the number of tanks and their status (i.e., adult mating age, juvenile, older than 1 year) and the number of total fish and tanks (Fig 14C, **top**). Because of limitations in graphing capabilities within FMPA, we have opted to generate reports using Google Charts Application Programming Interface (API), a third-party software (as outlined below in the Importing Water Quality section). Selecting the “Vitality Summary” button in this layout will display the number of births across a rack, room, or facility, with weekly, monthly, or yearly reports (Fig 14C, **bottom**). These graphical reports for facility stats and fish stats are both populated by user-entered data from dead fish reports, mating records, and tanks that are retired. In this “View Stats” layout, because different systems have unique headers and labels for data, we have simplified uploading external data. The management and partitioning of users and labs in FishNET, combined with barcode labeling, will allow the PIs and animal facility managers (or staff) to track animal usage and husbandry and enable accurate billing and IACUC reporting in real time. FishNET can also alert users if fish require attention or care.

**Fig 15:**
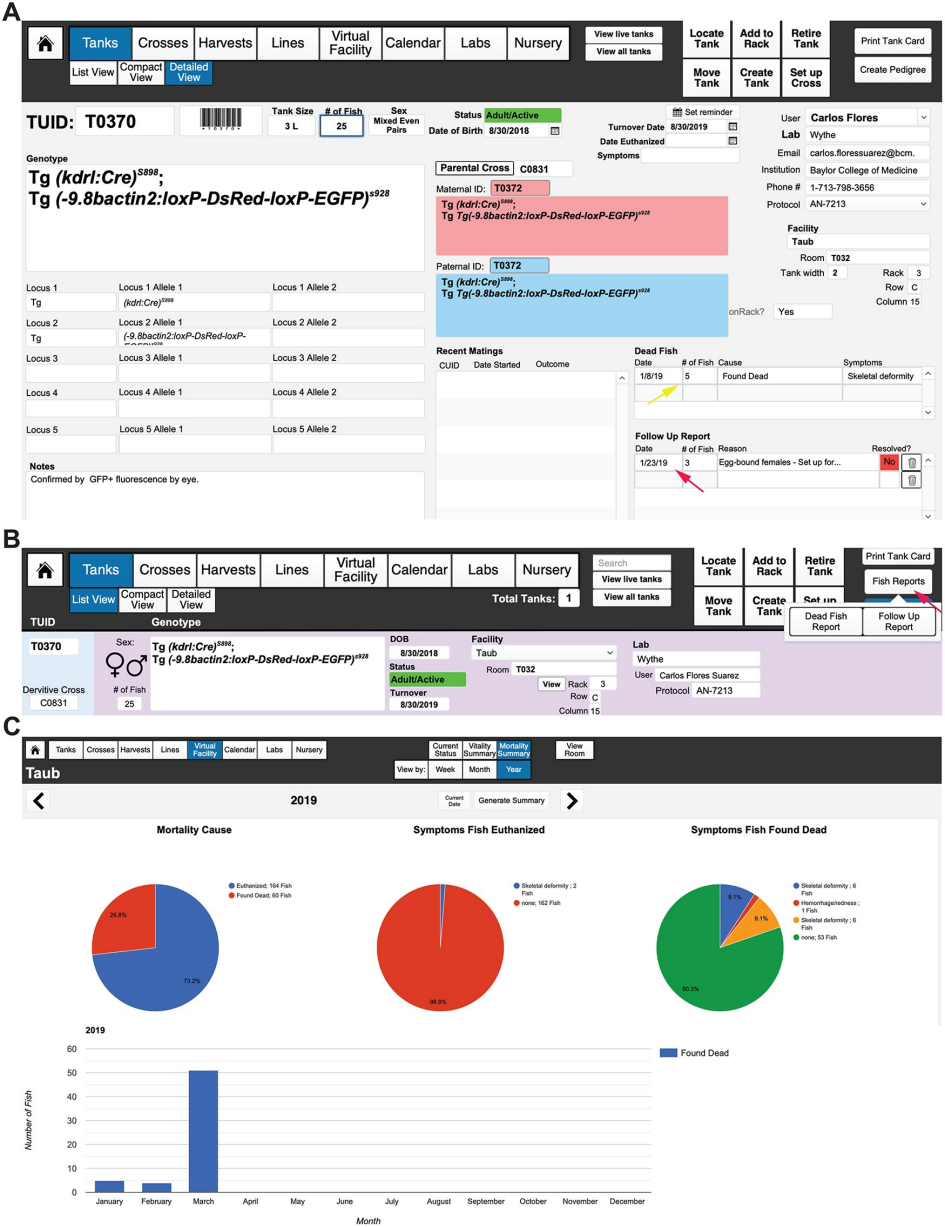
Generating follow-up reports with FishNET. (A) Individual fish within a tank can be brought to the attention of the tank owner and facility staff by creating a Follow-Up Report (indicated by the red arrow). A census of dead fish can be entered in the “Detailed View” of a tank (yellow arrow). (B) Both dead fish report and follow-up report list can be found in the List View Layout under Fish Reports (indicated with red arrow). A graphical overview can be found in the “Facility, Room, or Rack Statistics” under “Mortality Summary” (red arrow). (C) “Mortality Summary” shows pie charts with 1) a breakdown of Mortality Causes, 2) Symptoms of Fish Euthanized, and 3) Symptoms of Fish Found Dead. A bar graph of yearly, monthly, or daily dead fish is shown below as well.

A caretaker can create a follow-up report in a tank detailed view (Fig 15A). A list of all follow-up reports can be found in the tank list view by selecting fish reports (Fig 15B, indicated with red arrow). For laboratories running FishNET through FileMaker Server, it is possible to generate automated email reminders to users and/or facility administrators. If this feature is desired, the administrator must open the script workspace (S2A Fig, left red arrow). Here, find the script called “Send Follow-Up” (right red arrow), and within the script, select the configure button (yellow arrow). In the send mail option (S2B Fig), select “Specify” next to the simple mail transfer protocol (SMTP) Server option. A new window will pop up in which the user administrator must enter their lab or institutional SMTP Server information. For calendar reminders, this process needs to be repeated on the “Send Reminders” script (S2B Fig, right). Once that is finalized, the user needs to go to the Filemaker Server Admin Console (S2C Fig). Under Configuration/Script Schedules (red arrow), select Create Schedule (indicated with yellow arrow) and select Filemaker Script as Schedule Type and click on “Set Database” (S2D Fig, red arrow). In the new window, select FishNET, and then enter the administrator username and password (S2D Fig, right). From here, go to select script (S2E Fig) and pick “Send Follow-Up” from the list. Lastly, choose how frequently you would like to send facility users the emails. This process can be repeated for the calendar alerts by adding a new schedule and selecting the “send reminders” script.

A graphical overview can be found in the “Mortality Summary” in the “Stats” category in the Facility, Room, or Rack layout (Fig 15C, left). Once a report is generated in the mortality summary, three pie charts give an overview of the cause and symptoms present in the fish, followed by charts with the total number of dead fish. This report can be generated weekly, monthly, or yearly. A weekly report that can be printed can be found in the “Tank List” view by selecting “Fish Reports/Dead Fish Reports” (S3A Fig). The user can select the facility and the dates to be included in the report (S3B Fig). Furthermore, a complete list of dead fish can be found in the “Dead Fish List” in the “Virtual Facility Room” list.

### Importing water quality records

**Fig 16:**
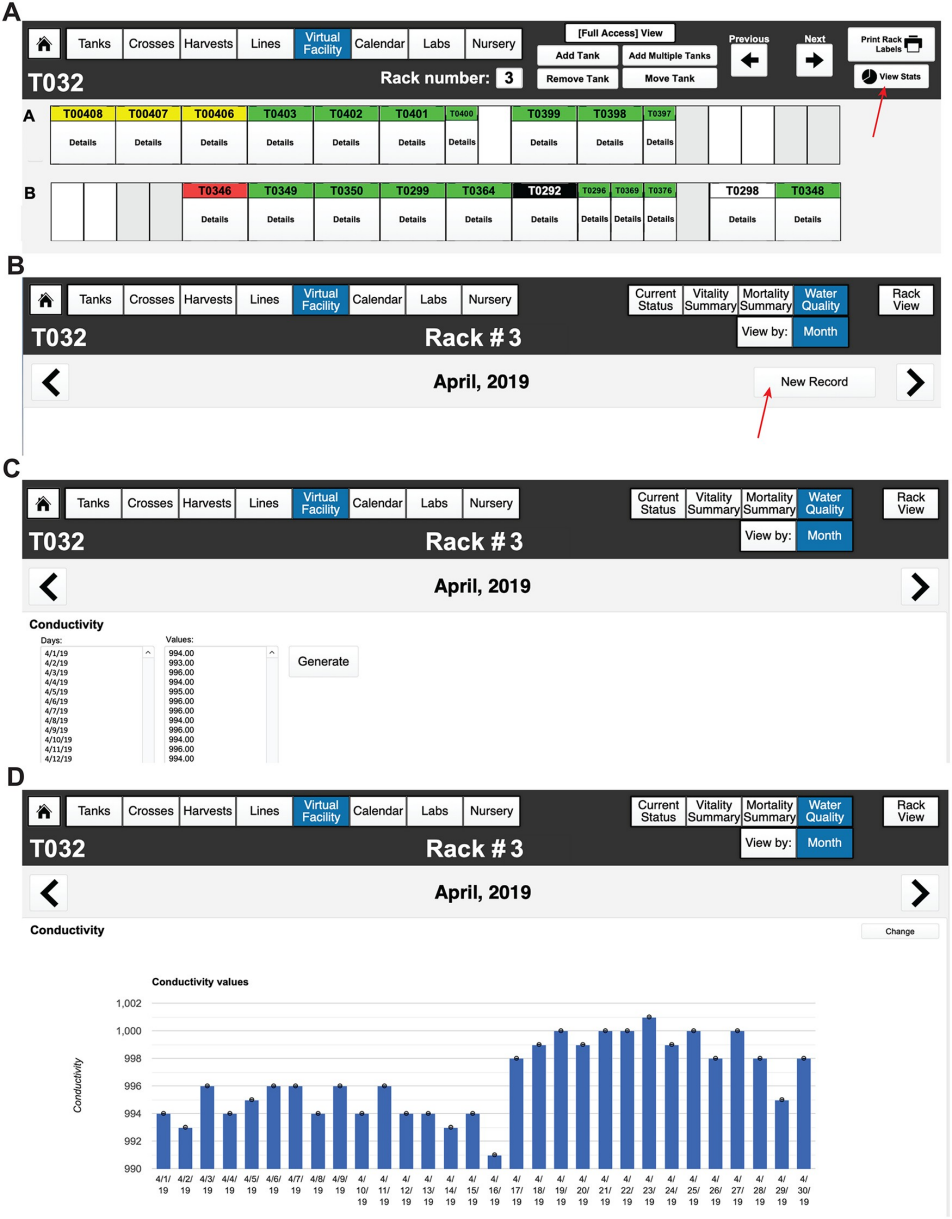
Importing water quality records. (A) Example of virtual rack view, to see rack statistics, select View Stats (shown with red arrow) on the top right corner of the screen. (B) Scroll through the months with the arrows in each side of the header. Once in the current month, select “New Record” (shown with red arrow). (C) Days and values can be pasted in the two fields; FishNET can show either a single value per day (conductivity) or the average of multiple readings a day. (D) FishNET uses Google Charts to display data, and for that reason, an internet connection is needed to use the graphing functions. Once the user is done adding the days and values, select “Generate.” Graphs can be modified by selecting “Change.”

We have enabled FishNet to generate water quality reports (e.g., conductance, pH, etc.) using the interactive Web service, Google Charts. Because of the limited options for data display in FileMaker, we have instead used Google Charts to generate graphical reports because it is more flexible in terms of graphing options, colors, and data input options. To generate these visual reports, export existing system water quality data as a .CSV file from a rack or facility of interest. Then, open FishNET and navigate to “Virtual Facility” and the specific “Room” containing the rack that the data correspond to. Next, select “Virtual View” for the individual rack of interest, then, in the next window, select “View Stats” (Fig 16A). Once in the individual rack stats view and in the present month of interest for the data, select “Water Quality Report” and “create new record” to import data (Fig 16B). Two different fields with either “Days” or “Values” will appear for each parameter field (conductivity, pH, temperature, nitrites, nitrates, and chlorine). From the .CSV file, simply copy the date of data acquisition (dd/mm/yy, or just the day number) in the first field and enter the values in the second field (making sure that the number of days and values are correctly related to one another). If there are multiple values recorded on one day, the system will display a bar graph with the average values and corresponding error bars.

### Printing tank, nursery, and rack labels

During the course of optimizing FishNET, we tested numerous printer configurations to find a sturdy, waterproof readhesive label that is also barcode compatible in a size usable with both large and small aquarium tanks as well as 10-cm petri dishes for fry. After much trial and error, we recommend using a BBP33 Brady printer and permanent, barcoded polyester labels for each tank location label on a rack. Unfortunately, the BBP33 printer only supports Windows computers. While some third-party vendors have developed BBP33 drivers for Mac, those we tested were unreliable. Instead, as a workaround, we suggest installing Windows 10 using Parallels Desktop for Mac (https://www.parallels.com/). As a cheaper alternative, one may use a Dymo 450 LabelWriter Turbo printer (Berkeley, CA, USA), but note that the labels are permanent and their removal may require scratching the tanks (details available, as well as printer layouts, upon request). Printing layouts for various labels are provided within FishNET under the main layout dropdown menu under the heading “Printing Layouts.” From here, users can visualize all of the printer layouts for petri dishes (for embryos), fry on the nursery, small tanks, large tanks, racks, and crosses. Each of these layouts are modifiable, and the label size can be changed to one that best meets a user’s needs, including which information should or should not be present on the labels. To create barcodes in FishNET, the Code 39 Barcode Font from IDAutomation (IDAutomationHC39M Code 39 Barcode.ttf) is required in any computer printing barcodes. It can be downloaded for free here: https://www.dafont.com/idautomationhc39m.font. Once the font is properly installed, the user needs to go to each printing layout (S4A Fig) and select “Edit Layout.” In edit mode, select the barcode rectangle and change its font from Arial to IDAutomationHC39M (S4B Fig). A barcode should appear. Then “Exit” the layout and “Save” changes. Repeat this process with each printing template.

After configuring a facility, within the “Virtual Facility” layout, a user can select their “Facility,” then select an individual “Rack,” and within this layout, a button to “Print Coordinates Labels” is present in the upper right area of the header. Selecting this function will create a pop-up window asking, “Which Row do you want to Print?” After entering the desired row of the rack you are currently using (e.g., “A”), then selecting “OK,” the labels for one row will be printed. For labeling the tanks on the main system and nursery, as well as the petri dishes containing embryos, we prefer to use a removable label, which has the added benefit of reducing adhesive buildup on tanks while also eliminating errors in transferring information (such as date of birth, genotype, sex, etc.) that occur during manual transcribing of labels. For an example of a completely labeled rack and tanks, see S4C and S4D Fig. To make input of tank locations easy, a barcode-compatible function is ready using the “Add Multiple Tanks” function in the Virtual Rack template, in which a sequential series of pop-up messages will prompt the user to scan the tank barcode followed by the rack location barcode (printed above).

### Frequently asked questions

A common error in FMPA (at least within our team) is that after importing data or deleting a record, a user would like to reset the TUID, CUID, LUID, or NUID number to a previous value (to eliminate gaps in numbering and ensure consistency). To do so, select “File” in the FMPA menu, go to “Manage,” then select “Database,” and a pop-up window will appear. Within this pop-up window, go to the “Tables” section and double click the table you want to edit (e.g., “Fish Crosses”). Then, double click the unique identifier Field Name (e.g., “CUID”), and a pop-up window with options for the field will appear. Then, select the “Auto-Enter” section. Here, the “Serial Number” option will have a checkmark, and below this option, the next serial value is defined (e.g., “C0032” if you are in “CUID”). To change this next value, simply edit the number after the first character (e.g., change “C0032” to “C0031” if you needed to delete one erroneously created cross record/CUID). After the changes are made, click the “OK” button to close the “Field Options” pop-up window, then select “OK” again to close the “Manage Database” window, and then finally save the changes.

## Discussion

Despite the increasing demand to ensure rigor and reproducibility at each step of the research endeavor, a robust, affordable, and intuitive archival database for zebrafish animal husbandry records has not been developed and widely adopted by the zebrafish community. We have created a facile, network-accessible relational, open-source database that meets the needs of researchers, animal husbandry staff, and institutional animal oversight committee members alike that creates and preserves comprehensive, detailed records for an individual lab or entire zebrafish facility. Additionally, such centralized, comprehensive record-keeping and the data visualization tools contained within FishNET will limit unnecessary duplication of lines within and across laboratories, ensure timely line turnover, and should flag fish husbandry or facility-wide issues (e.g., water quality, decreased fecundity, etc.) in real time, allowing for institutions to reduce the overall number of animals required for experiments and save critical research dollars.

Using the open-source, non-coding-based FMPA platform, the underlying architecture of FishNET can be modified by any end user to meet their unique needs or directly expanded upon to increase database functionality. FishNET also scales according to user demand, as does the FMPA platform, enabling functionality for research groups as small as one laboratory or as large as an entire institution. Future updates to FishNET will be available at http://www.wythelab.com/wythe-lab-databases. In regard to migrating data to updated versions of FishNET, FileMaker Pro offers a cost-effective data migration script package (FileMaker Data Migration Tool), available with the FileMaker Developer Subscription (https://store.filemaker.com/product/FDS) for $99 a year. Additionally, several third-party solutions are available online as well, although we have not yet validated any such software solution. Continual upgrades, along with integration of improved FMPA software and computer hardware technologies, will ensure that this inventory system continually evolves to meet the needs of the zebrafish community.

## Supporting information

S1 Fig Automated genotype annotation. A) Overview of a genotyping protocol. Expected products, primers, and thermocycling conditions, along with a PCR example and external document, can be imported. B) AppleScript editor settings window with a Script Menu activated. C) Genotyping gel in Adobe Photoshop CC 2017 with the Image Size windows showing the new width (4 in) and resolution (300 pixels/in) needed for proper gen annotation. D) Annotated gel imported in FishNET; the list of resources to which the gel is linked to are listed on the right. E) Example of an individual resource detailed record (H0002-1) with annotated genotype shown on the right.
(TIF)

S2 Fig Printing labels with barcodes. A) Label for crosses shown, with the layout path to get to all printing layouts (selected in blue). B) Edition of barcode field to activate the newly installed IDAutomationHC39M font. Once the field is selected, just change the font and exit layout, saving the changes. C) Photograph of labeled rack. D) A magnified view of the a few tanks and one row of a rack. Note the individual labels on the tanks, which contain relevant information (TUID, age, sex, genotype, lab owner, IACUC protocol, and position), as well as the labels on the rack itself, which correspond to the fixed positions/configuration of that rack. Also note the unique barcodes on each tank and the rack, which facilitate easy identification and moving of tanks, as well as setting up crosses or other functionality. IACUC, Institutional Animal Care and Use Committee; TUID, tank unique identifier.
(TIF)

S3 Fig Setting up automated email notifications. A) Accessing the Script Workspace indicated by red arrow in the left. Right panel shows the “Send Follow Up” script being selected (red arrow) along with how to configure the SMTP address (yellow arrow). B) Specify… (red arrow) is selected, and new Outgoing SMTP Server address is updated in the yellow arrow. This can be repeated in the “Send reminders” script (right panel). C) FileMaker Server Admin console showing Configuration/Script Schedule (red arrow) where a new schedule is selected (yellow arrow). D) Schedule type is set to FileMaker Script with Schedule Name “Follow Up.” Database is selected (red arrow) and administrator account set up (right panel). E) Script is set to “Send Follow Up,” with the option of repeat the script (send the email reminders) daily (as shown in the middle panel). A new Schedule can be added to add the “Send reminders” script to activate calendar reminders. SMTP, simple mail transfer protocol.
(TIF)

S4 Fig Creating dead fish reports. A) “Tank List View” layout with arrow pointing to “Print Dead Fish Report.” B) Example of a weekly dead fish report (which can be printed out).
(TIF)

S1 Table Comparison between FileMaker Server and FileMaker Server Cloud Applications. (DOCX)

S1 File Empty FishNET FMPA file. FMPA, FileMaker Pro Advanced.
(ZIP)

S2 File AppleScript for genotyping gels. (SCPT)

S3 File Spreadsheet for importing lines. (XLSX)

## Abbreviations

API: Application Programming Interface
Cre: bacterial Cre recombinase gene
CRISPR/Cas9: Clustered Regularly Interspaced Short Palindromic Repeats/CRISPR-associated protein-9 nuclease
CUID: cross unique identifier
fmp12: FMPA type
FMPA: FileMaker Pro Advanced
GAL4: the yeast Gal4 transcriptional activator
gCaMP: green fluorescent protein, calmodulin, and M13 peptide
HUID: harvest unique identifier
IACUC: Institutional Animal Care and Use Committee
LCD: liquid crystal display
LED: light-emitting diode
loxP: locus of X(cross)-over in P1
LUID: line unique identifier
NA: not applicable
NUID: nursery unique identifier
PHP: personal home page
PyRAT: Python-based Relational Animal Tracking
SMTP: simple mail transfer protocol
SQL: structured query language
SSL: secure sockets layer
Tol: transposable element
TUID: tank unique identifier
txt: Tab-delimited text
UAS: upstream activation sequence
VGA: visual graphics array
ZFIN: zebrafish information network
