# Supplementary figures and images for: FishNET: An automated relational database for zebrafish colony management
Source: PLoS Biol. 2019 Jun 20;17(6):e3000343. doi: 10.1371/journal.pbio.3000343 (PMC6605666; doi:10.1371/journal.pbio.3000343)

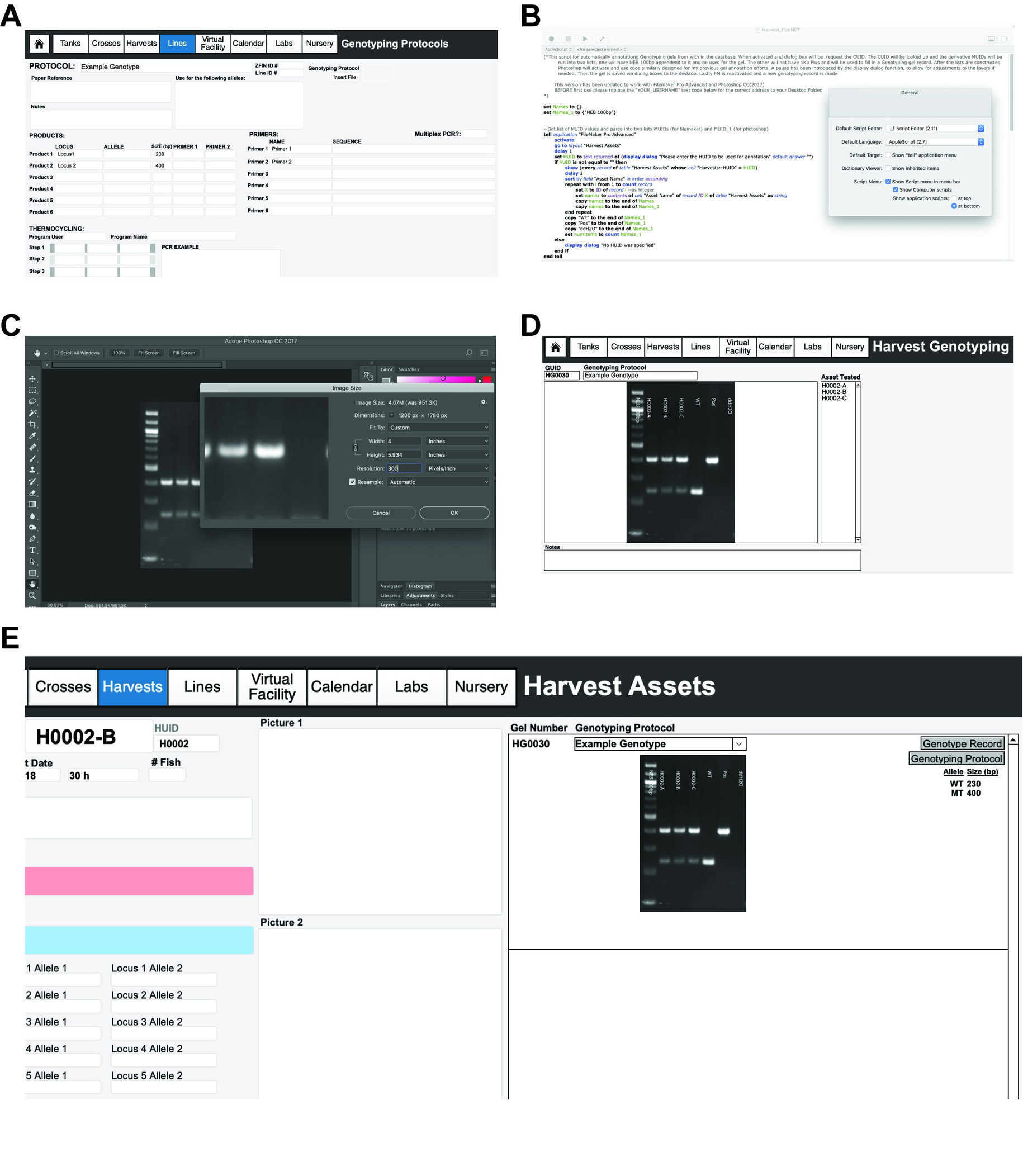

Supplement: S1 Fig — A) Overview of a genotyping protocol. Expected products, primers, and thermocycling conditions, along with a PCR example and external document, can be imported. B) AppleScript editor settings window with a Script Menu activated. C) Genotyping gel in Adobe Photoshop CC 2017 with the Image Size windows showing the new width (4 in) and resolution (300 pixels/in) needed for proper gen annotation. D) Annotated gel imported in FishNET; the list of resources to which the gel is linked to are listed on the right. E) Example of an individual resource detailed record (H0002-1) with annotated genotype shown on the right. (TIF) [file pbio.3000343.s001.tif]

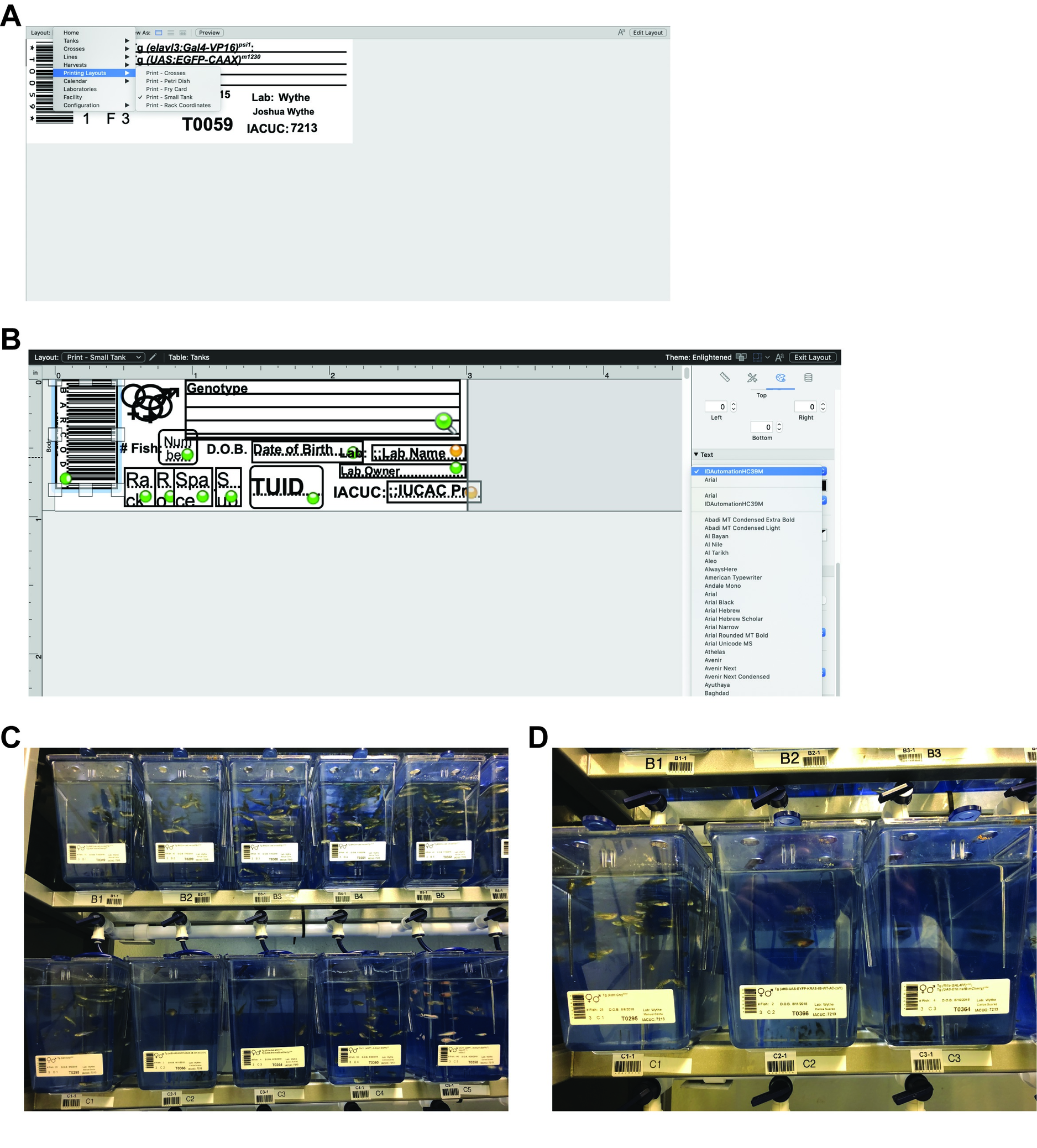

Supplement: S2 Fig — A) Label for crosses shown, with the layout path to get to all printing layouts (selected in blue). B) Edition of barcode field to activate the newly installed IDAutomationHC39M font. Once the field is selected, just change the font and exit layout, saving the changes. C) Photograph of labeled rack. D) A magnified view of the a few tanks and one row of a rack. Note the individual labels on the tanks, which contain relevant information (TUID, age, sex, genotype, lab owner, IACUC protocol, and position), as well as the labels on the rack itself, which correspond to the fixed positions/configuration of that rack. Also note the unique barcodes on each tank and the rack, which facilitate easy identification and moving of tanks, as well as setting up crosses or other functionality. IACUC, Institutional Animal Care and Use Committee; TUID, tank unique identifier. (TIF) [file pbio.3000343.s002.tif]

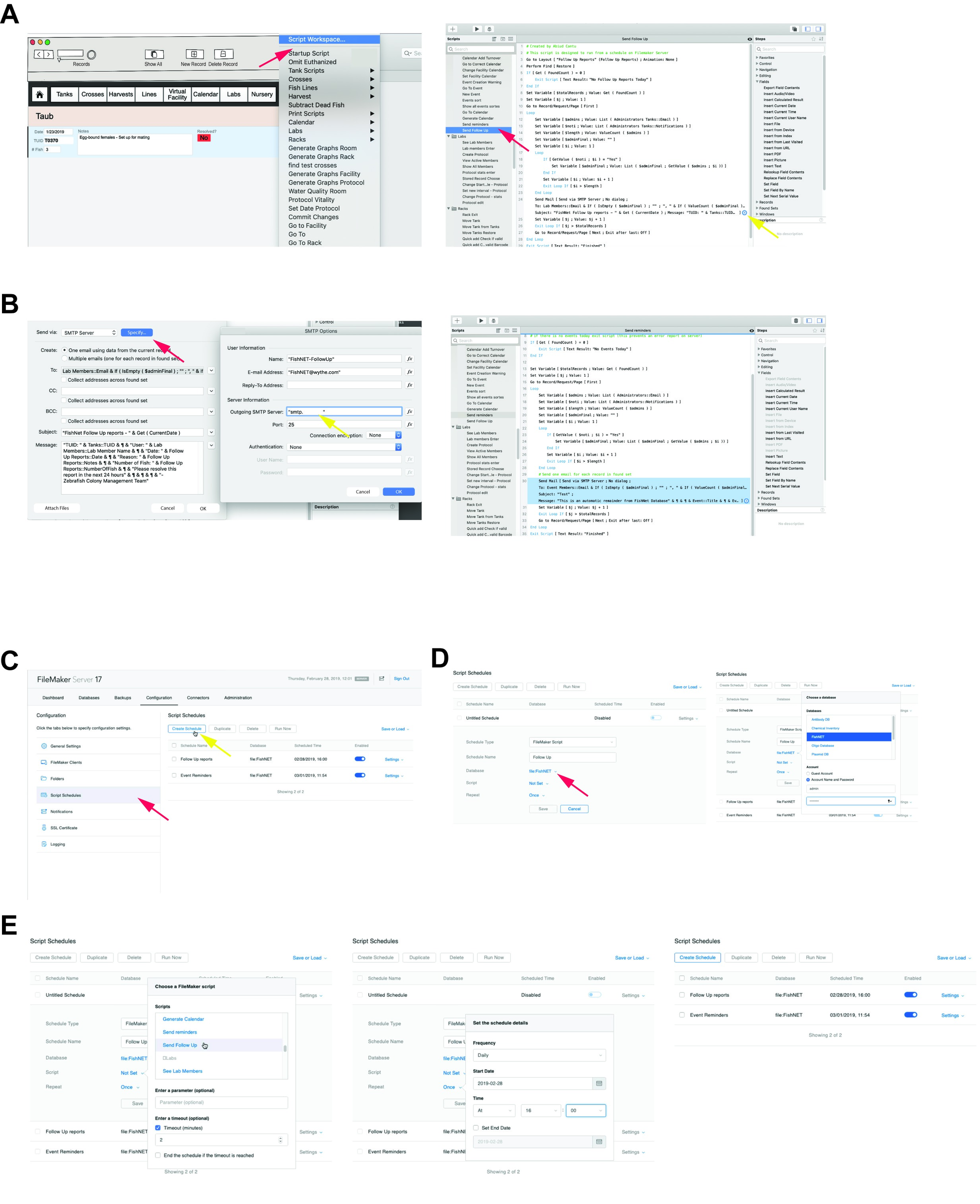

Supplement: S3 Fig — A) Accessing the Script Workspace indicated by red arrow in the left. Right panel shows the “Send Follow Up” script being selected (red arrow) along with how to configure the SMTP address (yellow arrow). B) Specify… (red arrow) is selected, and new Outgoing SMTP Server address is updated in the yellow arrow. This can be repeated in the “Send reminders” script (right panel). C) FileMaker Server Admin console showing Configuration/Script Schedule (red arrow) where a new schedule is selected (yellow arrow). D) Schedule type is set to FileMaker Script with Schedule Name “Follow Up.” Database is selected (red arrow) and administrator account set up (right panel). E) Script is set to “Send Follow Up,” with the option of repeat the script (send the email reminders) daily (as shown in the middle panel). A new Schedule can be added to add the “Send reminders” script to activate calendar reminders. SMTP, simple mail transfer protocol. (TIF) [file pbio.3000343.s003.tif]

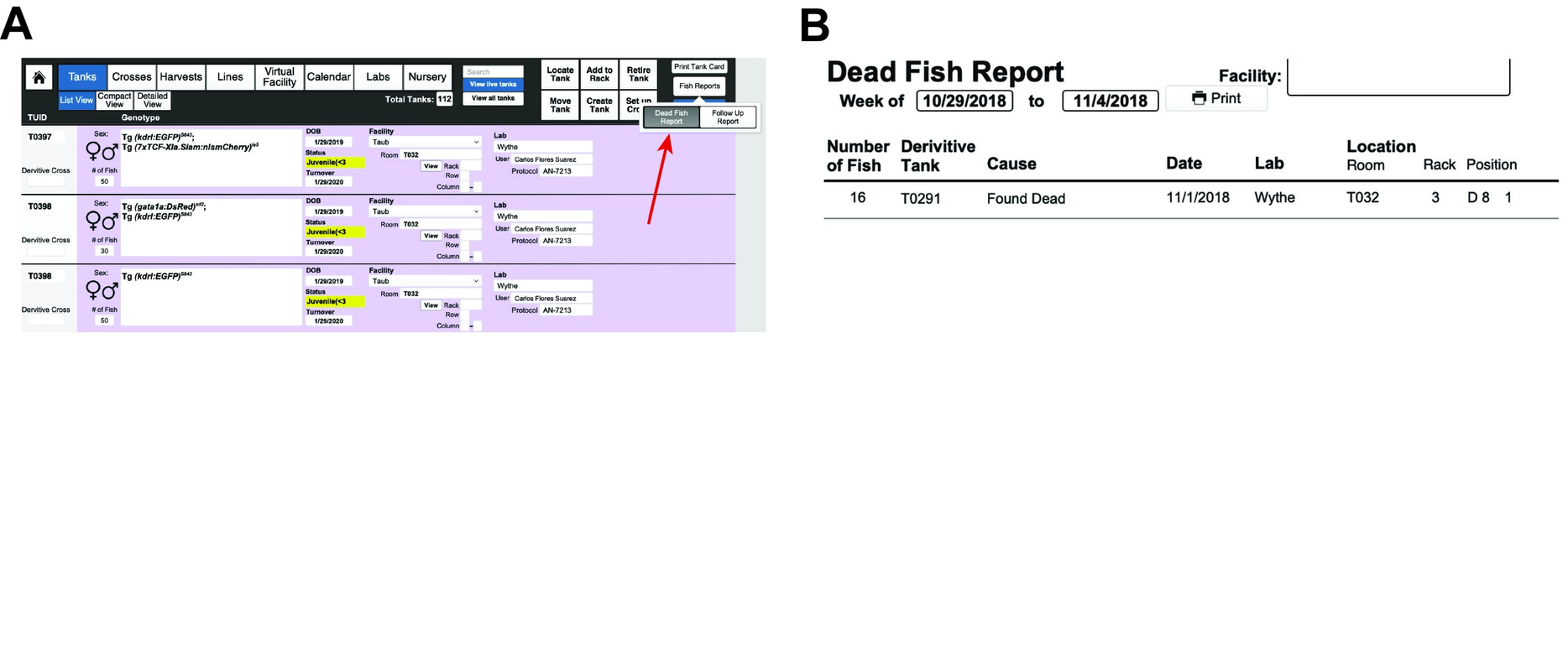

Supplement: S4 Fig — A) “Tank List View” layout with arrow pointing to “Print Dead Fish Report.” B) Example of a weekly dead fish report (which can be printed out). (TIF) [file pbio.3000343.s004.tif]
